# The optical nose: Monolayer sensitization of Au surfaces for plasmonic gas sensing

**DOI:** 10.1126/sciadv.aea1478

**Published:** 2026-04-08

**Authors:** Elle W. Wyatt, Sarah May Sibug-Torres, Marika Niihori, James W. Beattie, Tabitha Jones, Nicolas Spiesshofer, Jana Hofmann, Bart de Nijs, Jeremy J. Baumberg

**Affiliations:** ^1^ NanoPhotonics Centre, Cavendish Laboratory, Department of Physics, JJ Thompson Avenue, University of Cambridge, Cambridge CB3 0US, UK.; ^2^ Physics for Sustainable Chemistry Group, Department of Physics, JJ Thompson Avenue, University of Cambridge, Cambridge CB3 0US, UK.

## Abstract

Robust real-time gas sensing is important for many fields, including agriculture and health care analysis of breath/biofluid volatiles. Ammonia is ambiently present at parts per billion (ppb) to parts per million (ppm) levels, but current detection technologies suffer long measurement times, instability, cost, and issues with selectivity. Here, surface-enhanced Raman spectroscopy (SERS) is markedly improved through precision precleaning protocols, which allow surface sensitization of the metal facets using a water monolayer. Harnessing monolayer aggregates of densely packed gold nanoparticles with sub-nanometer spacing defined by rigid scaffolding molecules gives sub-ppm detection of ammonia at room temperature. Accessing the poorly studied high–wave number [>2500 per centimeter (cm^−1^)] region provides much improved discriminatory capabilities, enabling us to generalize this approach to a range of volatile organic carbon (VOC) molecules including ethanol, methanol, and acetone.

## Teaser

SERS of sub-ppm ammonia is improved through precision surface sensitization of Au nanoparticle aggregates using a water monolayer.

## INTRODUCTION

The ability to detect gases and volatile organic compounds [volatile organic carbons (VOCs)] at low concentration is of interest in fields from safety, air quality monitoring, climate mitigation, and agriculture to health care (*1*–*6*) relevant for COVID, asthma, and diabetes. In most cases, gases and VOCs are present at between parts per million (ppm) and parts per billion (ppb) concentrations, which requires highly sensitive methods for detection.

Ammonia is an analyte of interest for breath analysis for certain types of cancer and kidney disease (present at a few hundred ppb) (*7*–*10*) as well as monitoring atmospheric pollution due to industry and agriculture (irritant effects begin at ~10 ppm) (*11*–*14*). The current gold standard for detection is gas chromatography–mass spectrometry (GC-MS), which is highly sensitive (ppb) (*15*) but has high complexity, size, and cost (*16*–*18*). Aside from GC-MS, current methods of ammonia monitoring are commonly based on electrochemical resistance changes in metal oxides or polymer films, achieving limits of detection (LODs) of 0.1 to 100 ppm (*19*–*21*). These approaches also suffer from issues with selectivity, changes in humidity, and long-term stability or reversibility of interactions.

Surface-enhanced Raman spectroscopy (SERS) can offer highly specific and sensitive information about analyte molecules absorbed at a surface (*22*, *23*), including the analyte structure and its interactions with the surface. SERS can thus deliver an in situ, inexpensive sensing methodology with high sensitivity and specificity for trace gas and VOC detection without the need for preconcentration. SERS has recently gained traction for measuring VOCs using substrates from lithographically fabricated to colloidal nanoparticles, but SERS studies of ammonia detection are not widespread, with previous works focusing mainly on liquid conditions (*24*, *25*). These studied the Raman peaks of ammonia and those originating from ammonia-water interactions but report SERS detection in solution only down to LOD_soln_ ~ 1 ppm. Raman spectroscopy of gaseous ammonia has been reported at different temperatures when diluted in nitrogen but without any details on the concentrations (*26*).

We recently developed a reproducible SERS substrate that exploits the self-assembly of gold nanoparticles (AuNPs) into two-dimensional (2D) close-packed near-monolayer aggregate arrays (“MLaggs”) with precision (0.9 nm) plasmonic nanogaps defined by cucurbit[*n*]uril (CB[*n*], *n* = 5 to 8) molecular spacers (or “scaffolds”) (*27*–*29*). Crucially, these substrates can be precision precleaned to remove all organic surfactants and contaminants in their production, and additional cleaning cycles can regenerate the MLagg sensing surface between uses (*27*, *28*). Even under ambient conditions, a monolayer of water is seen on the AuNP facets (*30*). Large SERS enhancements (*28*) (>10^6^) for molecules in the gaps between AuNPs make MLaggs ideal for sensing low concentrations of analytes, including in the gas phase. We previously demonstrated SERS sensing of several VOC molecules from the headspace of a volatile in the fingerprint region of 600 to 1800 cm^−1^ using cucurbit[5]uril (CB[5])–scaffolded MLaggs (*27*). In the current work, the spectral regions are crucially expanded to include < 450 cm^−1^, where interactions with gold are seen, and >2800 cm^−1^, where C─H, N─H, and water O─H peaks arise. With this increased spectral information, the mechanisms that mediate the MLagg ammonia and VOC sensing can be understood.

In contrast to liquid-based sensing, the water monolayer at the nanoparticle surfaces advantageously acts as a surface sensitization layer for gas sensing using MLaggs. Ammonia vapor interacts both with this water monolayer, as well as directly with the Au, allowing detection down to a few ppb. Using other nanogap scaffolds and solvents tunes or blocks these interactions. Surface sensitization is also demonstrated for other small VOCs (alcohols and ketones) in the ppm range, heralding the realization of an “optical nose” (*31*). This clarifies debates about whether vapor sensing is optimized with direct gas adsorption onto a sensor or when predissolved into a solvent and sensed in the liquid phase (*32*).

## RESULTS

**Fig. 1.**
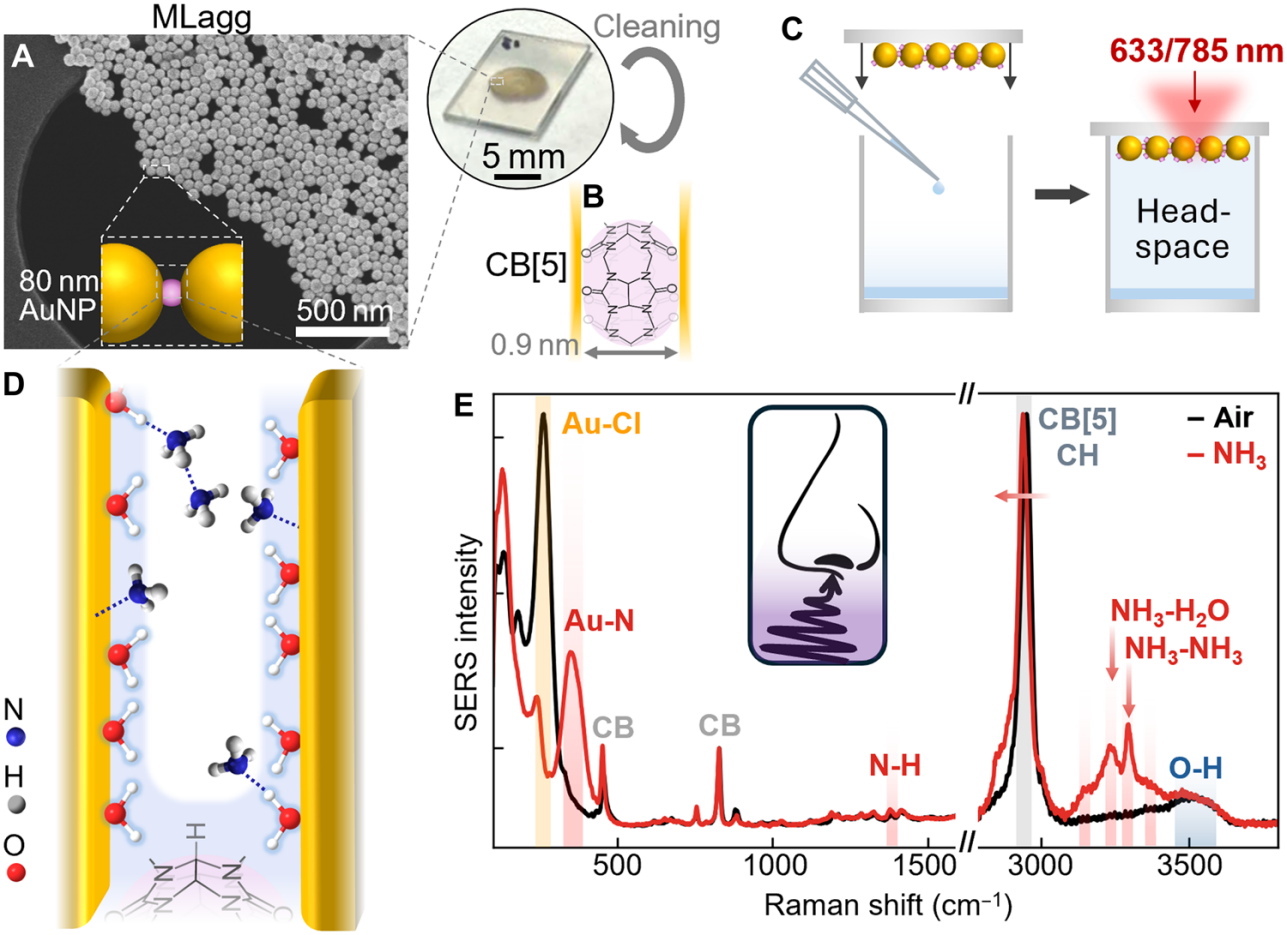
Multilayer aggregates (MLaggs) for ammonia vapor sensing. (**A** and **B**) AuNPs are aggregated with CB[5] and deposited on a coverslip to form a dense film with (B) precisely defined nanogaps. (**C**) For headspace sensing, 400 μl of 28% ammonia solution is added to a glass vial, which is then sealed with an MLagg-coated coverslip and left for 5 min for the volatile to saturate the headspace. (**D**) Schematic of the nanogap, identifying different ammonia interactions with the water monolayer and with the Au facets. (**E**) SERS spectrum of the MLagg in air (black line) and in ammonia vapor (red line), showing the appearance of peaks characteristic to ammonia.

Pristine ultraclean AuNP surfaces are formed within immobilized MLaggs by plasma oxidation, which removes all surfactants and CB[5] molecules and oxidizes the nanoparticle surfaces (Fig. 1A; Materials and Methods) (*27*). A subsequent rescaffolding step reduces the gold oxide, reinserting ligands to scaffold the gap, reforming precision 0.9-nm gaps with high enhancement factors (Fig. 1B). Volatile sensing is performed by placing small volumes of the compound in a sealed chamber, which evaporates and saturates the headspace (Fig. 1C and concentration calculations in section S2 and table S1). MLagg SERS spectra are taken through the supporting coverslip and compared to the spectrum of MLaggs in air (without an analyte).

For MLaggs rescaffolded with CB[5] and measured in a saturated ammonia atmosphere [red line; 450 parts per thousand (ppt)], new peaks appear compared to the same MLagg sample in air (Fig. 1, D and E, black line). Reference sharp peaks at ~450 and 826 cm^−1^ correspond to the carbonyl portals of CB[5] scaffold molecules and remain unchanged (their binding is unaffected). In the low–wave number region, a strong Au─Cl peak (from the cleaning step) is replaced by a peak at ~340 cm^−1^ [identified as Au─N in electrochemical cycling of ammonium compounds (*33*); see below].

Changes are also seen at high wave numbers near water O─H lines (~3500 cm^−1^) and CB[5] C─H lines (~2900 cm^−1^). In ammonia vapor, four additional peaks appear between 3000 and 3400 cm^−1^, two of which are sharper (3293 and 3231 cm^−1^). Previous Raman studies of ammonia in solution show that the N─H line at 3293 cm^−1^ comes from hydrogen-bonded NH_3_─NH_3_, whereas the N─H line at 3231 cm^−1^ is hydrogen-bonded NH_3_─H_2_O (*24*, *25*, *34*). This latter must form on the water monolayer, with additional NH_3_ molecules interacting with the first NH_3_ layer (Fig. 1D). Computational studies of ammonia absorption on Au agree that NH_3_ can bind either directly to Au atoms or via water molecules absorbed on the surface (*35*). As well as these new lines, a substantial red shift in the dominant CB[5] C─H line (of Δν = −12.5 cm^−1^) and an emerging C─H line at 2865 cm^−1^ arise, indicating that ammonia changes the CB[5] molecular environment. Similar effects are also seen for CB[6,7]-scaffolded MLaggs in NH_3_ vapor (section S1 and fig. S1). Other interactions may occur outside nanogaps but, being outside the SERS hotspots, they are not sensed. These SERS data show that the response of MLaggs to NH_3_ vapor is a complex mixture of interactions with the scaffolding CB[5] molecules, the water monolayer in the Au nanogaps, and direct interactions with the Au facets.

**Fig. 2.**
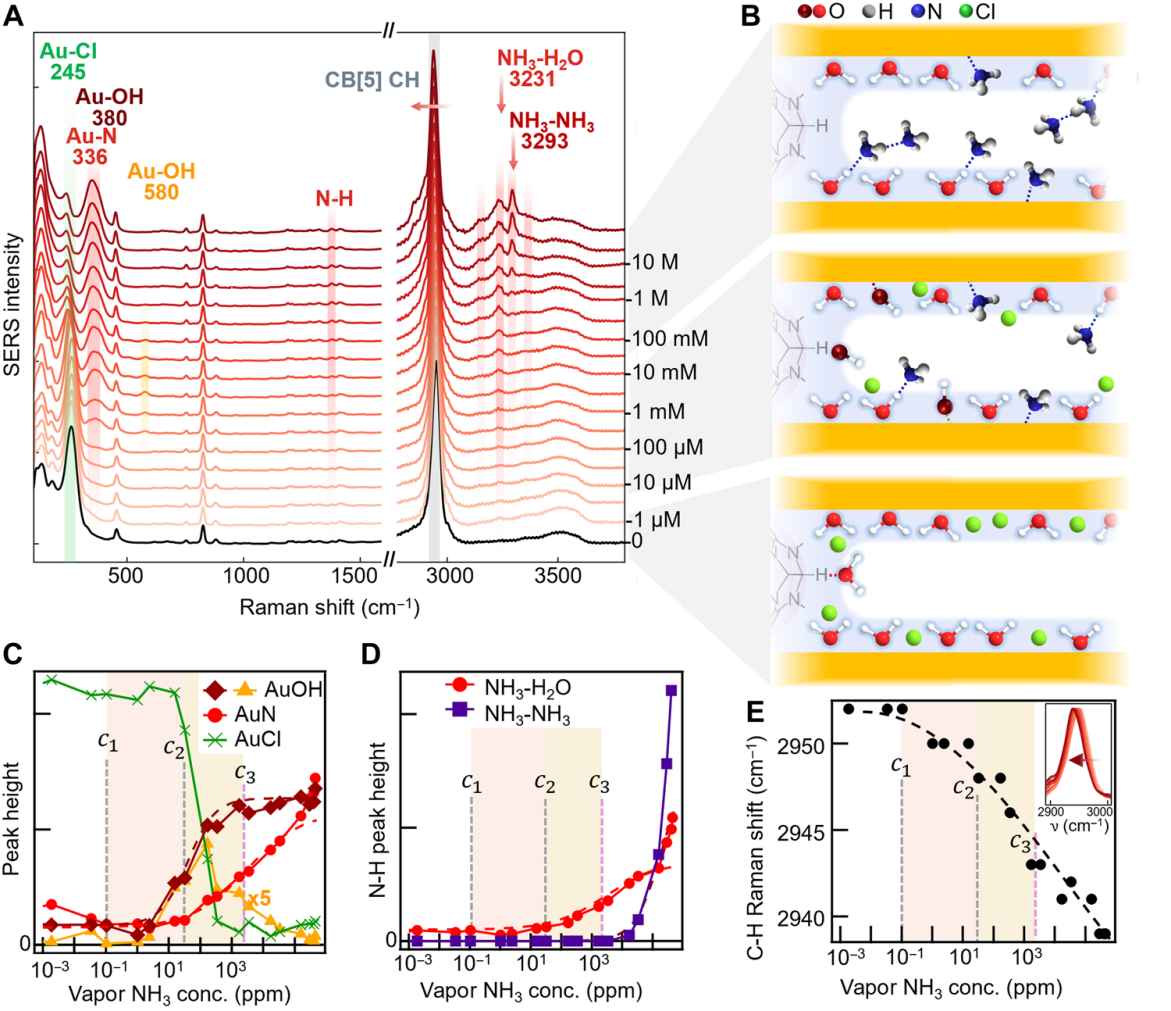
Headspace concentration series for ammonia sensing. (**A**) SERS spectra for different added ammonia solution concentrations, with changing peaks shaded. Corresponding headspace concentrations are 1 μM = 2 ppb, 10 μM = 110 ppb, 100 μM = 2.4 ppm, 1 mM = 32 ppm, 10 mM = 340 ppm, 100 mM = 3.5 ppt, 1 M = 35 ppt, and 10 M = 290 ppt (see table S1). (**B**) Schematic configurations in different ammonia sensing regimes identified in concentration series: Initial binding of NH_3_ displaces Cl^−^ ions and forms Au─OH, followed by the second layer of NH_3_ molecules at higher concentrations. (**C** and **D**) Extracted peak heights for fits (dashed line) and concentration thresholds $c_{1-3}$ (see Results section). (**E**) Spectral shift of the CB[5] C─H stretch versus NH_3_ concentration.

To further understand NH_3_-MLagg interactions, a headspace concentration series is measured for CB[5] MLaggs, by serially diluting the ammonia solution in the headspace (concentration calculations in section S2, fig. S2, and table S1). As the NH_3_ vapor concentration increases from 0 ppb to >400 ppt, gradual changes appear in the MLagg SERS spectra (Fig. 2). Even for low headspace NH_3_ concentrations, it is found that the SERS signal saturates within 5 min so the headspace system is at equilibrium (fig. S3).

Characteristic SERS peaks for NH_3_ appear in the ppb range, whereas above $c_{1}$ ~ 100 ppb the CB[5] C─H peak starts shifting to lower energies (Fig. 2, A and E). These effects can be seen in more detail by fitting the peak areas (section S3). Above 100 ppb, two close-spaced peaks (individually identified in fig. S4) at 336 cm^−1^ (Au─N) and 380 cm^−1^ [Au^(0)^─OH] grow (Fig. 2, A, C, and D) with the latter appearing first and the former rising once Au─Cl is removed. At the same time, a Au^(I)^─OH peak at 580 cm^−1^ appears (*36*), reaching maximum intensity at ~100 ppm.

These data suggest how species rearrange on the AuNP surface, with the Au─Cl first displaced by formation of Au^(0)^─OH (Fig. 2C, diamonds) and Au^(I)^─OH (triangles). Fits to the Langmuir-Hill model (dashed line) with $S\left(c\right)=S_{i}{\left[1+{\left(K/c\right)}^{p}\right]}^{-1}$ give dissociation constant K and Hill coefficient p for each species. For both Au─OH lines, we find $K_{\text{Au}-\text{OH}}$ ~ 40 ppm $\equiv c_{2}$ , and $p_{\text{Au}-\text{OH}}$ ~ 1 (indicating uncorrelated binding).

The ppb-induced red shifts in the C─H line suggest that OH^−^ (produced by adding NH_3_) hydrogen bonds to the equatorial proton of CB[5] through the dissociation equilibrium NH_3_ + H_2_O ↔ NH_4_^+^ + OH^−^ (Fig. 2B, middle) with p*K*_a_ = 9.25. This also provides OH^−^, which displaces Cl^−^ ions on Au, maximizing at 100 ppm.

The Au─N signal from NH_3_ binding (Fig. 2C, circles) rises more slowly ( $K_{\text{Au}-\text{NH}3}\equiv c_{3}$ ~ 3000 ppm and $p_{\mathrm{Au}-\mathrm{NH}3}$ ~ 0.5). Because [OH^−^], [NH_4_^+^] $\propto {\left[{\text{NH}}_{3}\right]}^{1/2}$in this domain (fig. S2), this accounts for $p_{\text{Au}-\text{NH}3}$ = 1/2 if the rate-limiting binding step requires NH_4_^+^. Exactly the same rise in the NH_3_─H_2_O signal is seen (Fig. 2D, circles), implying that NH_4_^+^ is similarly involved. Because the dynamic range Δlog conc≃4/(pln10) (see section S4 and fig. S5), this range is enhanced by $10^{1/p}$ = 100-fold compared to normal binding. The fall in the Au^(I)^─OH line occurs at the same rate, implying that Au─N binding replaces the Au─OH. This is also consistent with the point at which the concentration of NH_3_ exceeds that of OH^−^ in solution (fig. S2).

At even higher concentrations, the high–wave number N─H peak from NH_3_─NH_3_ rapidly rises (Fig. 2D, squares) with $K_{\text{NH}3-\text{NH}3}$ ~ 200 ppt and $p_{\text{NH}3-\text{NH}3}$ ~ 1. This implies that, once NH_3_─H_2_O coverage is complete, a second NH_3_ layer rapidly forms over the first (Fig. 2B, top). The continuous red shift of the main C─H peak over the whole concentration range (as well as the growth of three shoulder peaks at 2860, 2900, and 3000 cm^−1^) implies that the CH and CH_2_ groups on CB[5] interact with the surrounding ions. Of key importance is that these effects span a concentration range of >5 orders of magnitude, a far wider sensor dynamic range than is typical while not suffering from drift (fig. S3). However, we note that, due to this complex range of spectral features and peak shifts, it is not straightforward to use the traditional 3σ analysis to calculate a statistically robust LOD while accounting for all the effects seen. Independent gas-standard calibration with flow measurement should enable this to be quantified in the future.

These effects are highly reproducible when performed for different MLagg samples (three repeats exactly superimpose; fig. S6A), which is contingent on the precleaning step. Headspace sensing using deuterated solutions (section S5 and figs. S7 and S8) also shows expected red shifts. Comparing Au─Cl and Au─N peak heights gives relative standard deviation (RSD) between repeats of ~5%, which is noteworthy in this field (*37*).

Comparing vapor (Fig. 2) and liquid sensing (fig. S9) from the same 100 μM NH_3_ solution show that the headspace Au─N peak is five times larger compared to in solution (similar for the N─H peak). This shows that bulk water competes for nanogap binding sites, thus reducing sensitivity and emphasizing the need for humidity control in precision sensing. It suggests that monolayer water forms a good compromise between direct vapor binding versus presolvation in water.

**Fig. 3.**
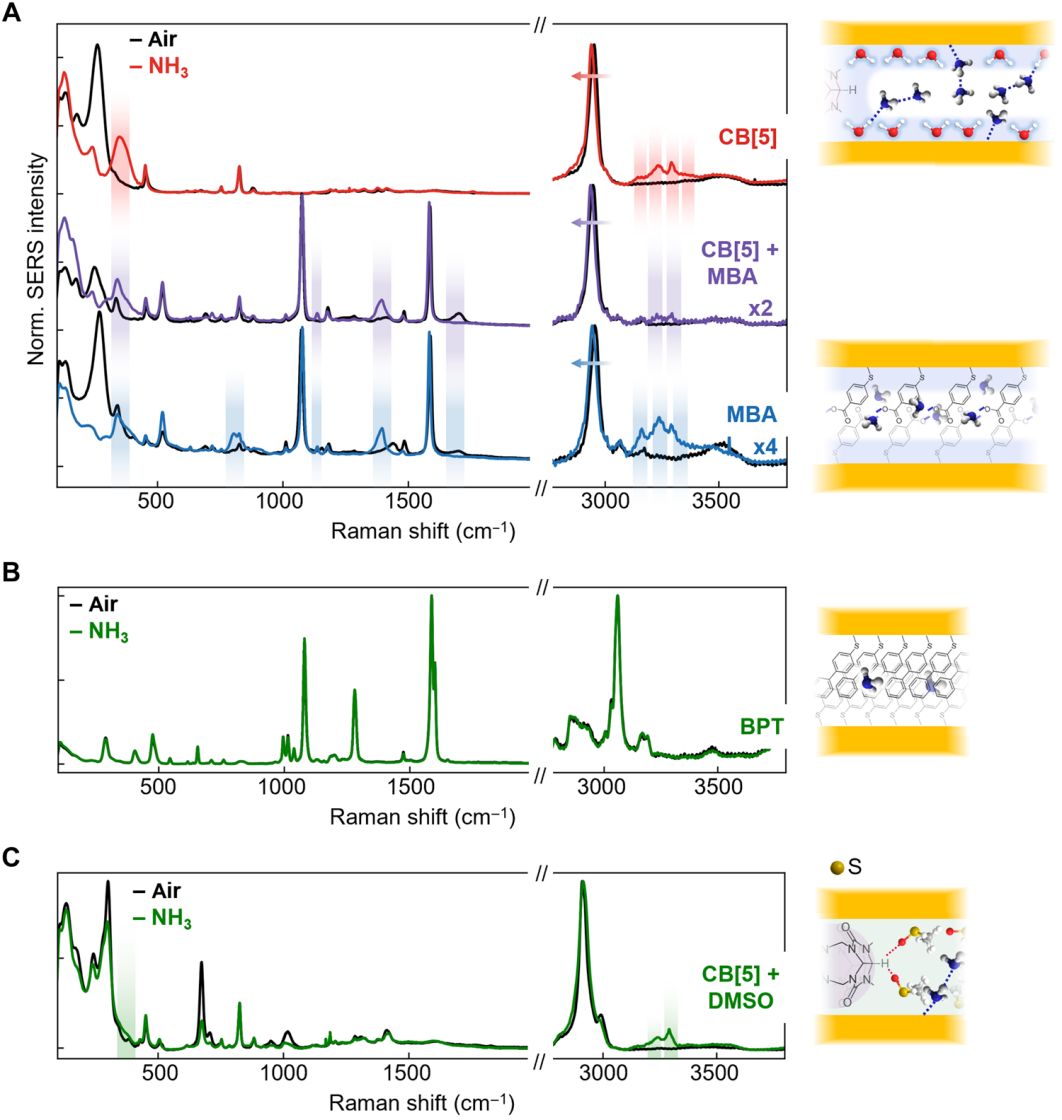
Effect of different scaffold molecules and nanogap solvents. (**A**) SERS of MLaggs rescaffolded with CB[5], CB[5]-MBA, and MBA in air and in a 10 M ammonia headspace, peak changes indicated. (**B**) MLaggs rescaffolded with BPT show minimal peak shifts on ammonia binding. (**C**) Displacement of the water monolayer by DMSO reduces ammonia interactions in the nanogaps. Schematics suggest the possible interactions of ammonia molecules in the nanogaps.

Apart from monolayer water acting as a surface sensitizer, rescaffolding provides the opportunity to tune MLagg nanogap chemistry through choice of a rescaffolding ligand. Thiols strongly bind to Au, removing the gold oxide after plasma cleaning without HCl (*38*). We thus first use 4-mercaptobenzoic acid (MBA), a common SERS pH indicator (*39*). Comparing MLaggs with CB[5] and/or MBA (Fig. 3A) shows that direct binding of NH_3_ to Au is strongly inhibited by the dense packing of thiol molecules. In addition, NH_3_-induced deprotonation of the MBA is seen by signals switching from 1700 cm^−1^ (COOH stretch) to 1400 cm^−1^ (COO^−^ stretch). Although effects of NH_3_ are observed down to ~100 ppb (section S6 and fig. S10), this nonspecific (de)protonation is not a signature unique to ammonia (*40*).

Another approach is to eliminate water from the nanogaps, here using hydrophobic biphenylthiol (BPT) (removing O─H lines at ~3500 cm^−1^; Fig. 3B, black line). In NH_3_ vapor, only minimal shifts in BPT peaks (<2 cm^−1^) and intensity ratios are seen, and no N─H peaks or Au─N appear (again due to dense thiol packing). A number of other thiol scaffolds were also tested for ammonia sensing (fig. S11), showing either large SERS changes due to protonation and hydrogen bonding as for MBA [e.g., mercaptopyridine (MPY)] or minimal intensity changes and peak shifts similar to BPT [aminothiophenol (ATP) and nitrothiophenol (NTP)]. Similarly, sensitivity is suppressed by soaking CB[5]-scaffolded MLaggs in dimethyl sulfoxide (DMSO) for 30 min, replacing most surface water even after drying (Fig. 3C, black line) (*30*, *41*). Although headspace NH_3_ displaces ~70% DMSO, the Au─N peak is 10-fold weaker than without DMSO. The high–wave number spectrum also changes, with a weaker NH_3_─DMSO peak. Because DMSO is aprotic, NH_3_ no longer ionizes and OH^−^ peaks are absent.

Optimal NH_3_ detection combines a water monolayer and CB[5] nanogap scaffold. We note that, even at our lowest concentrations of ~2 ppb, a background NH_3_ signal is observed [see also principal components analysis (PCA); fig. S6], which we believe is due to ambient NH_3_ (our LOD is below this). Precleaning is vital for the sensitization developed here. Using MLaggs without plasma cleaning plus rescaffolding (using NaCl or CB[5] aggregation) gives signals that are extremely weak (section S6 and fig. S12). Citrate remains on these AuNPs, preventing Au─N interactions just as for DMSO. Effective plasma cleaning and surface preparation are thus vital for plasmonic gas sensing applications.

**Fig. 4.**
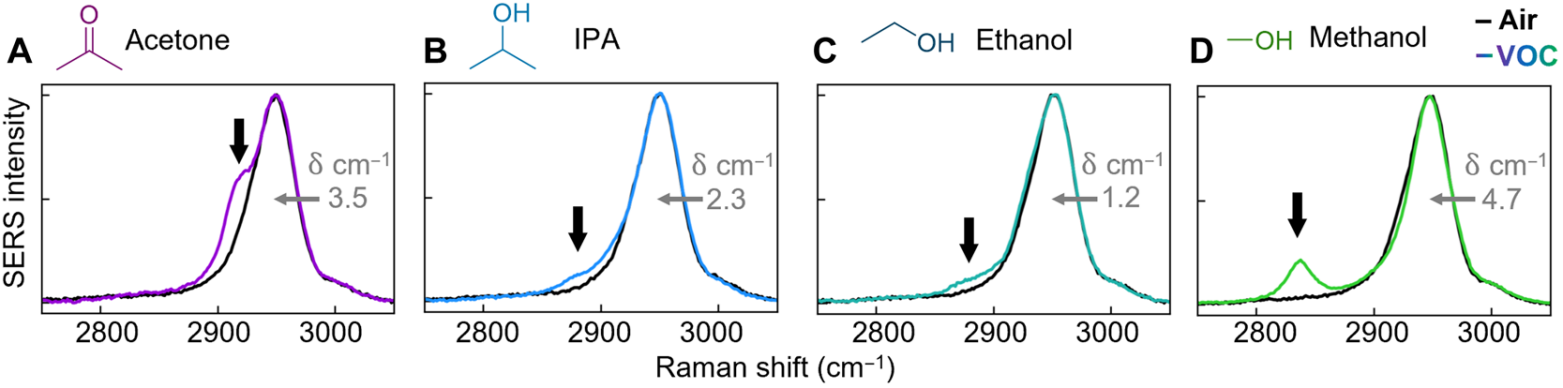
SERS sensitivity of C─H vibration to VOCs. SERS spectra for MLaggs in air (black lines) and in the headspace of four different VOCS at their undiluted saturation concentrations: (**A**) acetone (252 ppt), (**B**) IPA (44 ppt), (**C**) ethanol (81 ppt), and (**D**) methanol (1660 ppt), showing the appearance of C─H peaks characteristic to each (arrows). Peak shifts of CB[5] C─H lines as labeled to bring them into registry.

Using this scheme, we now sense other small VOCs relevant for breath/volatile analysis, which are challenging to discriminate in low-cost sensors (concentrations in table S2). Ethanol, methanol, and acetone were selected as commonly available VOCs, with changes in breath concentrations linked to diseases such as lung cancer or diabetes (*3*, *4*). We show improved sensitivities when including the high–wave number C─H and O─H peaks in headspace sensing. In all cases, characteristic analyte molecule peaks appear in the fingerprint region (C═O, C─O, and C─C; fig. S13) (*27*) along with additional lines between 2800 and 3000 cm^−1^ corresponding to C─H modes of each VOC (Fig. 4, A to D). Characteristic shifts are also seen in the CB[5] C─H peak from all VOCs.

Compared to NH_3_, these analytes produce a greater reduction in intensity and shift of the water monolayer O─H lines to a lower wave number [fig. S13; these O─H peaks correspond to a suppressed hydrogen bonding network (*30*)]. Decreasing O─H indicates that VOC molecules replace some of the water on AuNP surfaces. No clearly distinguished VOC O─H peaks are seen at ~3400 cm^−1^ for isopropyl alcohol (IPA), ethanol, and methanol, and nor is any significant VOC─Au bond seen.

Comparison of density functional theory (DFT) calculations of VOC Raman activity to the SERS spectra corroborate this mechanism (section S7 and fig. S14), matching the unique C─H high–wave number lines for each VOC. MLaggs effectively distinguish small volatiles by their C─H lines, using only a narrow waveband (2800 to 3000 cm^−1^). Large spectral separation ( λ ~ 775 nm) from 633-nm Raman lasers and low-cost longpass filters can deliver simple devices, which discriminate these VOCs.

Thiolated scaffolds similarly modify MLagg interactions with VOCs. For BPT-scaffolded MLaggs, sensing of acetone and methanol results in small but repeatable shifts of the major BPT lines (section S8 and fig. S15, A and B). Slightly larger effects are seen for methanol in a triphenylthiol (TPT)–scaffolded MLagg compared to BPT (fig. S15C). This implies that VOCs diffuse into densely packed molecular layers in the nanogaps and perturb their vibrations. Cysteamine-scaffolded MLaggs specifically respond to ketones over alcohols as bridging S─S interactions (*42*) are formed between neighboring cysteamine molecules. On exposure to VOCs, the S─S peak decreases for ketones (acetone and cyclopentanone) but not for alcohols (ethanol and methanol) (fig. S16A). This is due to hydrogen bonding between the cysteamine NH_2_ and the ketone C═O (*43*), which forces apart and weakens the S─S links (fig. S16B). The reduction in S─S intensity is related to the number of ketone molecules (30% reduction for 15 ppt cyclopentanone and 65% reduction for 252 ppt acetone). Choosing a nanogap scaffold thus gives selectivity for VOCs with particular functional groups (*44*).

## DISCUSSION

**Fig. 5.**
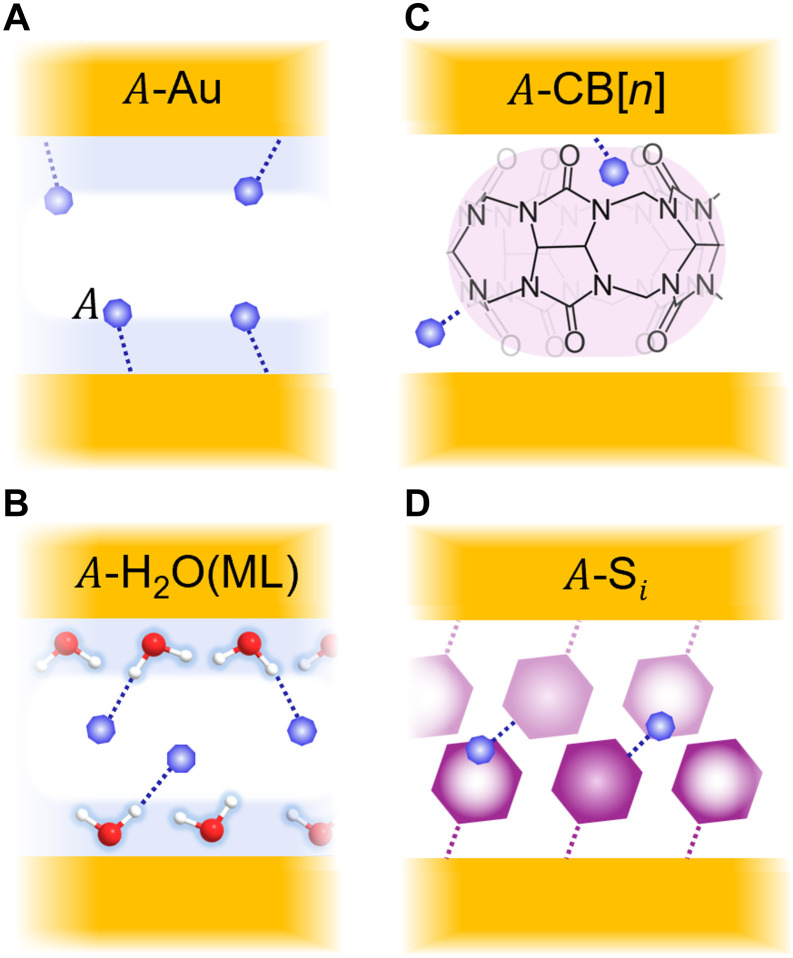
MLagg gas sensing schemes of analyte A. (**A**) Direct interaction with AuNP. (**B**) Interaction with the water monolayer (ML). (**C**) Interaction/sequestration by CB[*n*]. (**D**) Interaction with scaffold molecule (S*_i_*) functional groups.

We categorize VOC SERS sensing using precision plasmonic nanocavities depending on in situ interactions (Fig. 5). Analyte molecules ( A ) either interact directly with the AuNP surfaces, as for NH_3_ (Fig. 5A), or with the water monolayer on the Au facets, either by forming hydrogen bonds with the water molecules (as for NH_3_), or by partial displacement of the water layer (as for acetone) (Fig. 5B). Such interactions can be blocked when alternate sensitization layers (DMSO) or hydrophobic molecules are used to scaffold the nanogaps (such as for scaffolding $S_{i}$ = BPT). Binding interactions with scaffold molecules are also possible, to functional groups (as for cysteamine) or different molecule sizes (as for CB[*n* = 5 to 8]) (Fig. 5, C and D, and fig. S1). Multiplexed surfaces with differently functionalized gaps probed in parallel thus have great discriminatory power (*44*). Understanding these interfacial effects in detail for different VOCs is thus crucial, but for optimal sensor performance, other factors must also be accounted for. These include the effects of temperature and humidity on the Au surface and how to achieve precise humidity or solvent layer control, absorption of analytes into the Au surface [for instance, NH_3_ is not known to dissolve Au at room temperature (*45*)], and competitive binding effects in the nanogaps.

We have shown that precleanable plasmonic surfaces (MLaggs) sense ammonia down to ppb levels, achieving LODs matching GC-MS (~150 ppb) (*15*). In contrast to GC-MS, this does not require extensive sample preparation or long measurement times, enabling real-time and in situ measurements. As for GC-MS, Raman spectrometers can currently be bulky and expensive, but there has been much recent progress toward low-cost portable Raman instruments. MLagg interactions with gas molecules are mediated by the AuNP surface and its water monolayer, as well as the nanogap solvent and scaffold molecules. Precleaning/rescaffolding here avoids problems of drift often encountered for current gas sensors while providing quantitative calibration. The dynamics of this optical nose match human olfaction (currently <10 s; fig. S17) and potentially can be enhanced. For instance, suspending freestanding MLaggs over open apertures (as they are highly robust; Fig. 1A) allows alternative flow-through geometries that reduce diffusional distances <100 nm and reaction times potentially <1 ns given high flow rates.

These interactions can be generalized to other volatile amines, also of interest as VOC analytes (*4*, *10*). Further sensitization of AuNP surfaces is possible, either by using different molecules in the rescaffolding step or by altering the solvent in the nanogaps. This offers the potential to tune the MLagg affinity to different gases. These methods are applicable to a wide range of small VOC molecules, but for future applications, these must be extended to complex VOC mixtures as single VOCs will rarely be present in isolation. This could be achieved using selective MLagg surface sensitizations in different sensor regions to enhance differentiation of mixture components. Detection of VOC mixtures will also require more complex data analysis techniques involving machine learning to resolve overlapping SERS peaks from different VOCs, competitive binding effects, and large datasets. Classification of the observed gas sensing mechanisms demonstrates the versatility and quantitative reliability offered by precleaned MLagg systems, especially for machine learning modalities. Combining these developments opens up robust MLagg-based gas sensors for a diverse range of applications, with optimal surface functionalization chosen to enhance the selectivity and sensitivity of MLagg-based gas and VOC sensing.

## MATERIALS AND METHODS

### Monolayer aggregate preparation

Five hundred microliters each of chloroform (CHCl_3_) and commercial (BBI Solutions) citrate-capped 80-nm AuNPs was added to an Eppendorf tube. One hundred microliters of a 1 mM CB[5] solution was then added and shaken for ~1 min to initiate aggregation. The mixture was left to settle for the immiscible CHCl_3_ and aqueous phases to separate and the aggregated AuNPs to move to the interfaces (chloroform-aqueous and aqueous-air). The aqueous phase was washed with three 300-μl aliquots of deionized (DI) water to dilute the citrate salts and other supernatants and then concentrated by careful removal of the aqueous phase to form an ~5-μl aggregate droplet floating on the CHCl_3_. The droplet was deposited onto a precleaned sapphire coverslip (0.15 mm thick), which minimizes background fluorescence. Once dried, the resulting AuNP monolayer aggregate (MLagg) was rinsed with DI water and dried with N_2_.

The MLaggs were oxygen plasma cleaned for 45 min (oxygen mass flow: 30 standard cubic centimeter per minute; 90% radio frequency power) using a plasma etcher (Diener electronic GmbH & Co. KG) to remove CB[5], citrate, and other supernatants from the AuNP surfaces (verified using SERS). To reintroduce a scaffolding ligand, the MLaggs were immersed in 1 mM CB[5] solution prepared in 0.5 M HCl for 5 min and then rinsed with DI water and dried with N_2_.

To rescaffold the MLaggs with thiolated molecules, the samples were plasma cleaned for 45 min as before and then immersed for 30 min in a 10 mM solution of the thiol in ethanol.

All chemicals were purchased from Sigma-Aldrich, and aqueous solutions were prepared in DI water (>18.2 MΩ·cm^−1^; Purelab Ultra Scientific system).

### SERS measurements

SERS measurements were collected on a Renishaw InViva Raman confocal microscope, using a 20x objective (numerical aperture = 0.4) and a grating of 1200 lines mm^−1^. To collect the lower–wave number spectral regions, the 785-nm excitation laser was used in extended scan mode, with a 1-s integration time and 0.5% laser power (2.2 mW at the sample).

For MLaggs prepared with 80-nm nanoparticles, the plasmon resonance wavelength occurs at ~800 nm and hence SERS enhancement is optimal for 785-nm laser excitation (*27*). Use of an edge filter with cutoff at ~100 cm^−1^ enabled measurement between 100 and 450 cm^−1^.

A 633-nm laser was used to collect SERS spectra in the region of 2000 to 4000 cm^−1^, with a 30-s integration time and 10% laser power (0.37 mW at the sample). At these higher Raman shifts (2000 to 4000 cm^−1^), the SERS light collected by the spectrometer charge-coupled device (CCD) for 785-nm excitation would have λ > 1000 nm, where the CCD quantum efficiency is poor. Using a 633-nm excitation laser for these measurements shifts our detection light to shorter wavelengths, for better CCD detection efficiency and relatively good MLagg coupling.

All measurements were taken at room temperature, and spectra were calibrated with respect to silicon. Spectra were analyzed using the Python code, and any background was removed by iteratively fitting a polynomial to the base of the peaks. Static measurements were taken at 5 to 10 spots on the MLagg and averaged. Ten measurements taken on the same MLagg have an RSD of ~4%, calculated from the Au─N peak.

Examination of the Stokes: Anti-Stokes ratio indicates that these measurement conditions result in a MLagg temperature rise of <10 K mW^−1^ (*46*, *47*), and even after repeated exposure over 60 min, the water monolayer remains stable, with the O─H peak remaining at constant intensity (fig. S18) (*30*).

### Headspace sensing

A small volume (~400 μl) of each volatile compound was added to a 2-ml glass vial, and the upper rim of which had been coated with a layer of “Parafilm M.” The sapphire coverslip holding the rescaffolded MLagg was then gently pressed, MLagg side down, onto the top of the rim of the glass vial to create a sealed chamber. This was left for 5 min for the volatile vapor to saturate the vial environment, and then SERS spectra were taken through the glass coverslip. As the droplets of volatile compound in the vial never fully evaporate, it can be assumed that the analytes reach saturation concentration (see calculations in section S2 and tables S1 and S2).

To take a headspace ammonia concentration series, serially diluted ammonia solutions were used as the volatile compound, changing the partial pressure of the ammonia and hence the concentration in the headspace (whereas the water vapor concentration remained roughly constant at ~31 ppt; section S2). Measurements were made from low to high headspace concentrations. The MLagg sample was purged with an ~10-s N_2_ flow from a N_2_ gun before each subsequent measurement to remove any NH_3_ remaining from the previous measurement.

## Supplementary Materials

### This PDF file includes:

Sections S1 to S8 Figs. S1 to S18 Tables S1 and S2 References
