# Supplementary material for: The optical nose: Monolayer sensitization of Au surfaces for plasmonic gas sensing
Source: Sci Adv. 2026 Apr 8;12(15):eaea1478. doi: 10.1126/sciadv.aea1478 (PMC13060579; doi:10.1126/sciadv.aea1478)
Supplement: Supplementary file 1 — Sections S1 to S8 Figs. S1 to S18 Tables S1 and S2 References [file sciadv.aea1478_sm.pdf]

Science Advances
AAAS

Supplementary Materials for

The optical nose: Monolayer sensitization of Au surfaces for plasmonic gas sensing

Elle W. Wyatt *et al.*

Corresponding author: Jeremy J. Baumberg, jjb12@cam.ac.uk

Sci. Adv. **12**, eaea1478 (2026)
DOI: 10.1126/sciadv.aea1478

This PDF file includes:

Sections S1 to S8
Figs. S1 to S18
Tables S1 and S2
References

`

Section S1: Comparison of different CB[n]

Similar effects to CB[5] are also seen for CB[6,7] scaffolded MLaggs in NH_3 vapour (Figure S1). In both cases, the same broad Au-N band appears at ~362 cm^{-1}, though is less intense than for CB[5] when normalised to the CB[n] peaks (relative peak height decrease of ~40%) and is broadened to higher wavenumbers. Peaks also appear at 500 and 558 cm^{-1} (orange shading, Figure S1b), which are attributed to adsorbed OH on the Au surface (*36*). The Au-Cl peak at 250 cm^{-1} is less intense for the CB[6-7]-scaffolded MLaggs in air, suggesting the available area for direct interaction with Au is smaller, explaining their reduced Au-N sensitivity. Fewer Cl^- ions at the surface could also lead to the ~15 cm^{-1} shift of the Au-N peak for CB[6/7] compared to for CB[5], due to reduced vibrational Stark shifts.

With CB[6-7]-scaffolded MLaggs, high wavenumber peaks (3000–3400 cm^{-1}) are also similar to those for CB[5], although a slight shift in the NH_3-NH_3 peak for CB[7] MLaggs (by ~2cm^{-1}) is seen (Figure S1b). As for CB[5], the main C-H peak shifts with ammonia compared to air (Δv=-20 cm^{-1} for CB[6], -14 cm^{-1} for CB[7]), but the additional C-H peak appearing at 2865 cm^{-1} when in NH_3 becomes weaker as the gaps are scaffolded with larger CB[n] molecules. This could a size related effect, since the portal diameter for CB[5-7] increases with n (2.4, 3.9 and 5.4 Å respectively). It is possible that the larger CB[6-7] more easily accommodate NH_3 molecules at their portals close to the gold (both are known to form inclusion complexes with various aliphatic amines, with the amine group being situated close to the portal C=O of the CB[n]) rather than interacting with the equatorial C-H with CB[5] (Figure S1c). This may account for why in CB[6-7] some Au-N peaks shift to higher wavenumbers, and the effects of NH_3 on the 2865 cm^{-1} CB[n] C-H line diminish (Figure S1d).

`

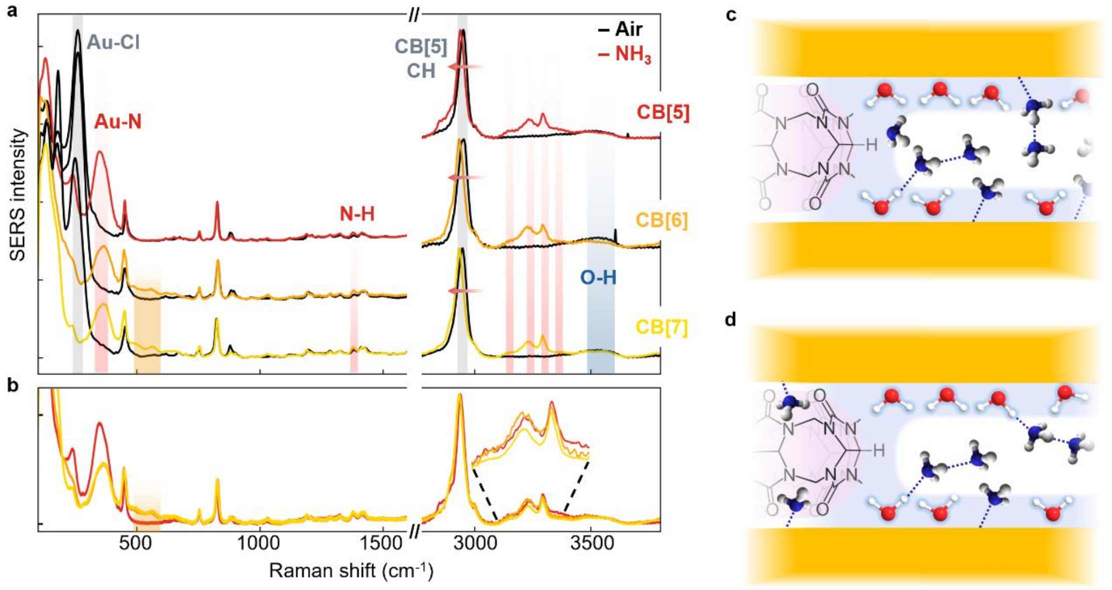

Figure S1. Comparison of ammonia sensing with CB[5-7]-scaffolded MLaggs. (a) SERS spectra from CB[5-7] MLaggs in air (black) and 450 ppth ammonia vapour (red: CB[5], orange: CB[6], yellow: CB[7]) showing appearance of peaks characteristic to ammonia. **(b)** Overlaid SERS from CB[5-7] MLaggs in ammonia vapour, scaled to show the peak differences clearly. **(c,d)** Schematics of nanogaps scaffolded with **(c)** CB[5] and **(d)** CB[6-7] to note suggested differences in ammonia interactions.

`

Section S2: Headspace concentration calculations

We first note that in contrast to solution concentrations, gas concentrations are always quantified in parts per million by volume or by mole (not by mass). Assuming full saturation is achieved (as the droplets of volatile compound in the vial never fully evaporate), the VOC partial pressure P in the headspace from solution concentration c is given by Henry's law as

$$P = c/H$$

where H is Henry's law constant and at lab temperature (20°C) is found for NH_3 to be H_{NH3}=27 M (*48*, *49*).

A range of concentrations can be achieved in this set-up by diluting the pure volatile liquid with water to change its partial pressure, where x is the molar fraction of the volatile liquid in solution. It was found that the concentration of water vapour in the headspace changes negligibly compared to the concentration of the analyte volatile compounds, due to its high partial pressure (~31 ppth).

However the aqueous concentration of NH3 in solution is not the same as that added, because of the equilibration between

$$NH_3 + H_2O \leftrightarrow NH_4^+ + OH^- \quad \text{(E1)}$$

$$H_2O \leftrightarrow H^+ + OH^- \quad \text{(E2)}$$

Using the added solution concentration c=[NH_3] + [NH_4^+], and the known equilibrium constants (*50*) $K_w = [\mathrm{OH^-}][\mathrm{H^+}]/[\mathrm{H_2O}]$=$10^{-14}$ and $K = [\mathrm{OH^-}][\mathrm{NH_4^+}]/[\mathrm{H_2O}][NH_3]$=$1.8 \times 10^{-5}$, allows the aqueous [$NH_3$] to be calculated at each c, by solving the cubic equation for $o = [\mathrm{OH^-}]$,

$$o^3 + Ko^2 - (cK + K_w) - KK_w = 0 \quad \text{(E3)}$$

Calculations of headspace concentrations are tabulated for the range of ammonia concentrations used (Table S1) and for the other VOCs (Table S2).

Table S1. NH_3 headspace concentration calculations. Volatile ammonia from diluted 28% solution.

Solution [NH_3] (M)	x	P (ppth, ppm, ppb)
17	2.35×10^{-1}	450 ppth
15	2.13×10^{-1}	408 ppth
10	1.53×10^{-1}	293 ppth
5	8.27×10^{-2}	158 ppth
1	1.77×10^{-2}	35 ppth
0.5	8.93×10^{-3}	17.2 ppth
0.1	1.80×10^{-3}	3.5 ppth
0.05	9.00×10^{-4}	1.7 ppth
0.01	1.80×10^{-4}	343 ppm
0.005	9.01×10^{-5}	170 ppm
0.001	1.80×10^{-5}	32 ppm
0.0005	9.00×10^{-6}	15 ppm
0.0001	1.80×10^{-6}	2.4 ppm
0.00005	9.01×10^{-7}	1.0 ppm
0.00001	1.80×10^{-7}	105 ppb
0.000005	9.00×10^{-8}	34 ppb
0.000001	1.80×10^{-8}	1.9 ppb
0	0	0 ppm

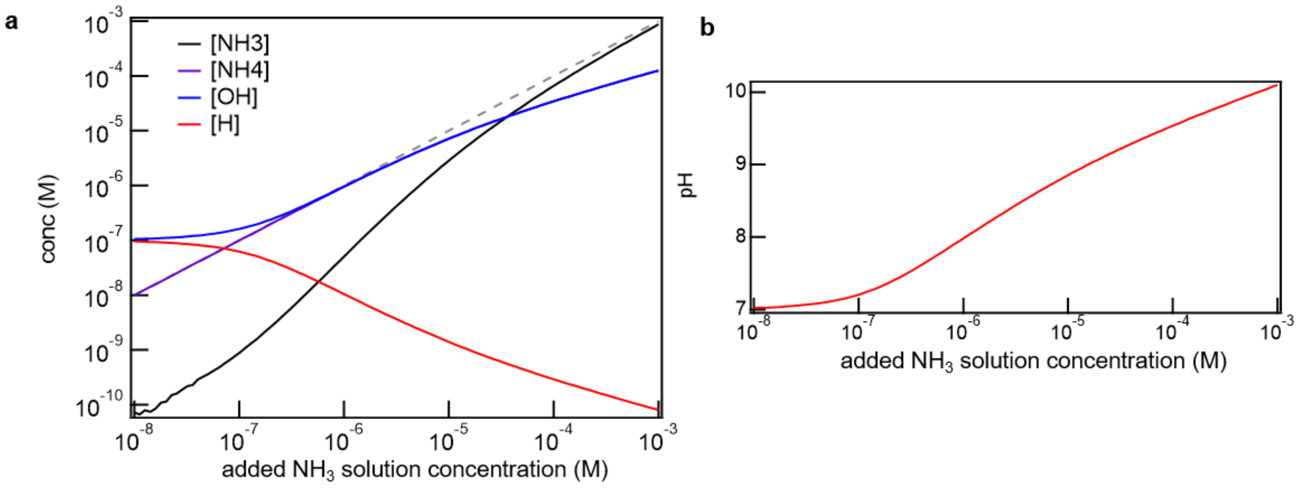

Figure S2. Concentration of species for different added ammonia concentration. (a) Final equilibrium solution concentrations of ammonia and water species when adding different solution [NH_3], see equation E3, and **(b)** resulting solution pH.

`

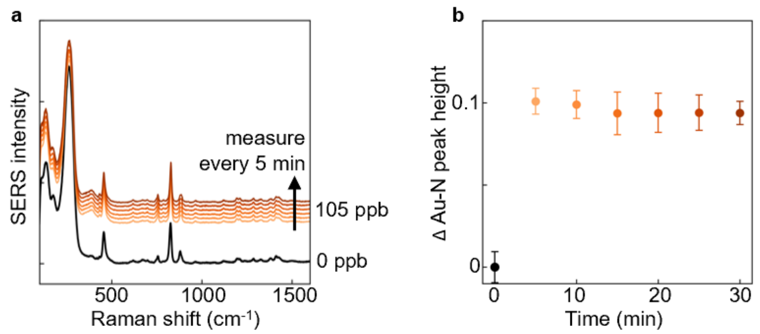

Figure S3. Saturation of the ammonia SERS spectrum. (a) SERS spectra measured every 5 minutes for a CB[5] MLagg in a 105 ppb NH_3 headspace. **(b)** Plotting the change in Au-N peak height shows no systematic changes after 5 minutes, showing the system is at equilibrium after 5 minutes, even at low headspace concentration.

`

Section S3: Peak fitting protocol

To fit the low wavenumber regions, a second order polynomial background was first fit to spectra. Then, the position and width of the 450 cm^{-1} CB[5] peak was fixed (from the 0 ppm spectrum) and Gaussians were fitted to the spectra for the Au-Cl and two Au-N lines (required for a good fit), constrained to be ~10 cm^{-1} around peak positions obtained by peak finding (using the high NH_3 concentration spectrum). The Au-OH peak was fit separately (Figure 2c, Figure S3a,b).

In the high wavenumber region, the water spectrum was first subtracted (approximated as the water spectrum in air, 0 ppm ammonia), and then four peaks were fit with positions constrained to be ~5 cm^{-1} around the peak positions fitted to the SERS spectrum at the highest ammonia concentration (Figure 2d, Figure S3c,d).

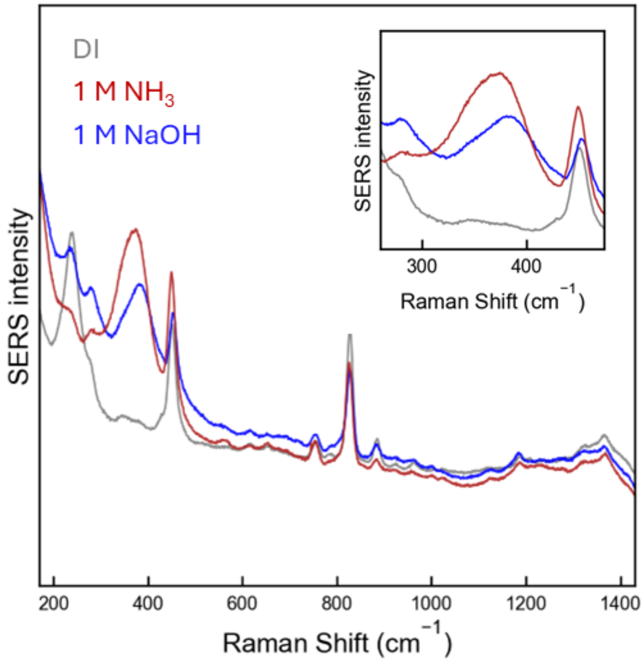

Figure S4. SERS of NH_3 and NaOH solutions. SERS spectra of MLagg-CB[5] in deionised water (DI, grey line), 1 M NH_3 (red line), and 1 M NaOH (blue line). Inset highlights the separate Au-N and Au-OH^- peaks at 336 and 380 cm^{-1}.

`

Section S4: Langmuir Isotherm fits and derivation

The conventional Langmuir isotherm vs concentration $[c]$ can be recast into a simple tanh function,

$$S([c]) = \frac{1}{1+\left(\frac{k}{[c]}\right)^{p}} \equiv \frac{1}{2}\left\{1+\tanh\left[\frac{p}{2}(\log[c]\ln 10 - \ln k)\right]\right\} \qquad \text{(E4)}$$

which makes it simple to fit the functional form $vs \log[c]$ as conventionally plotted, and to extract the limiting cases. Around the point of maximum growth (using the approximation $\tanh x \sim x$ for small x) this becomes

$$S([c]) = \frac{p}{4}(\log[c]\ln 10 - \ln k)$$

with gradient $\frac{\ln 10}{4}p \sim 0.58p = p/1.74$.

This gives an estimate for the concentration dynamic range (on a log scale) where this gradient at midpoint intersects the highest and lowest S as $\Delta\log[c] = 4/(p\ln 10)$.

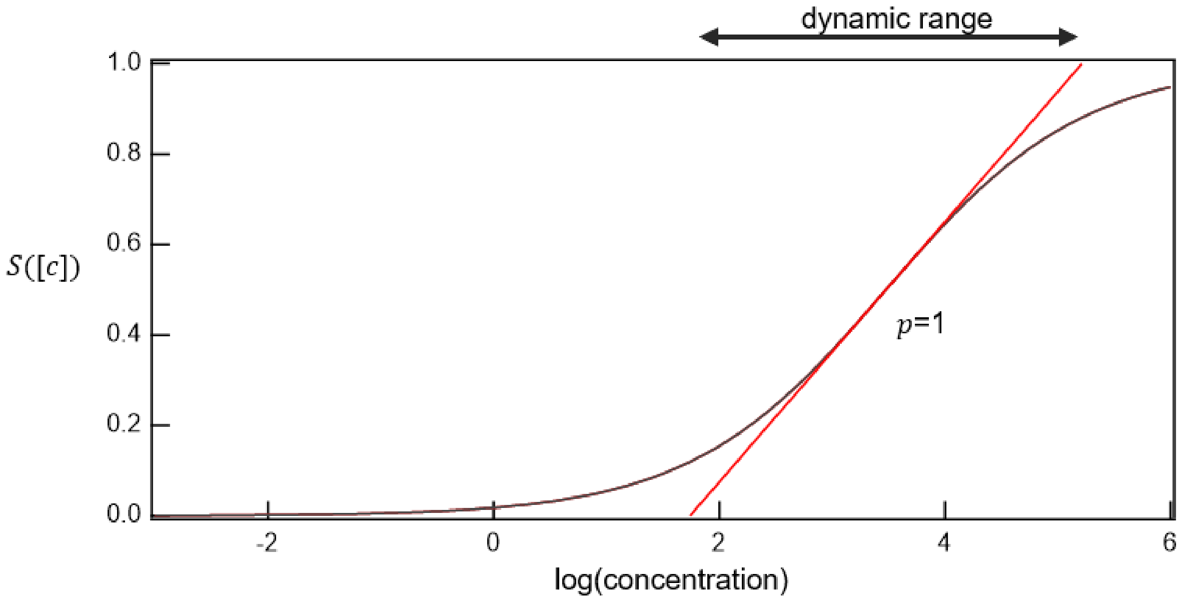

Figure S5. Langmuir isotherm estimate of concentration dynamic range. Full isotherm (equation E4), together with fit about detection midpoint (here for p=1), showing estimate of dynamic range from intersection of maximum and minimum signals.

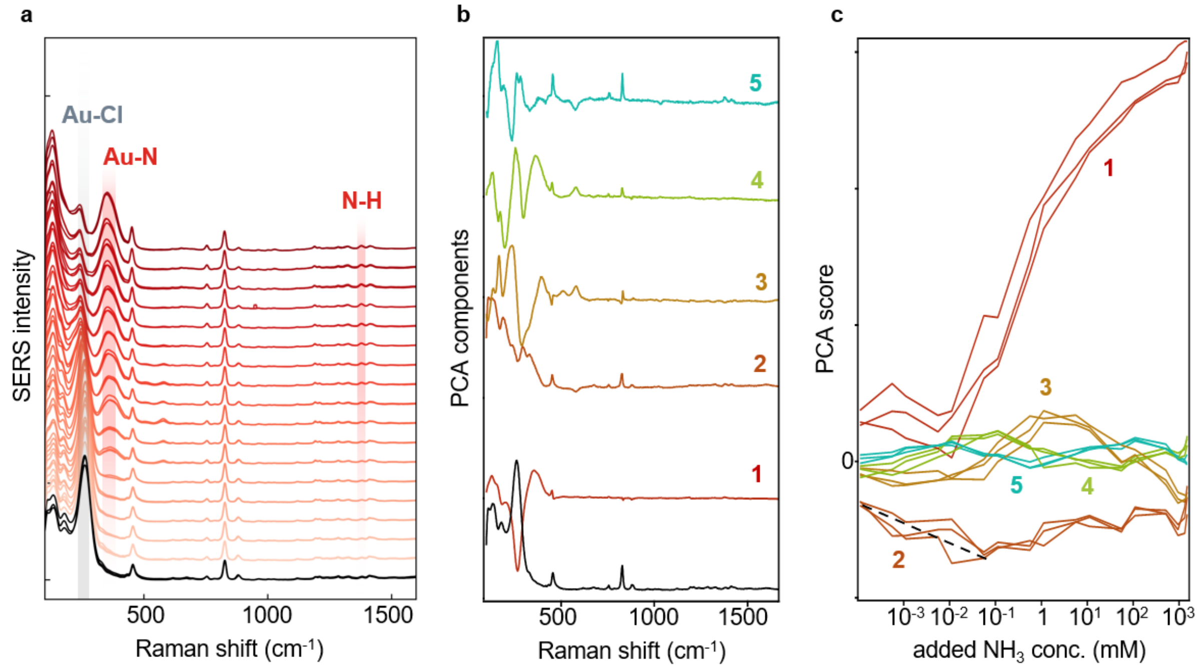

Figure S6. Reproducibility of ammonia sensing. (a) Overlayed concentration series for three different samples of CB[5] scaffolded MLaggs showing minimal difference in their SERS spectra at each concentration. (b,c) PCA analysis of these spectra splits the changes into (b) five distinct contributions with (c) different scores at each concentration of the ammonia solution.

`

Section S5: Comparison to deuterated ammonia and solution sensing

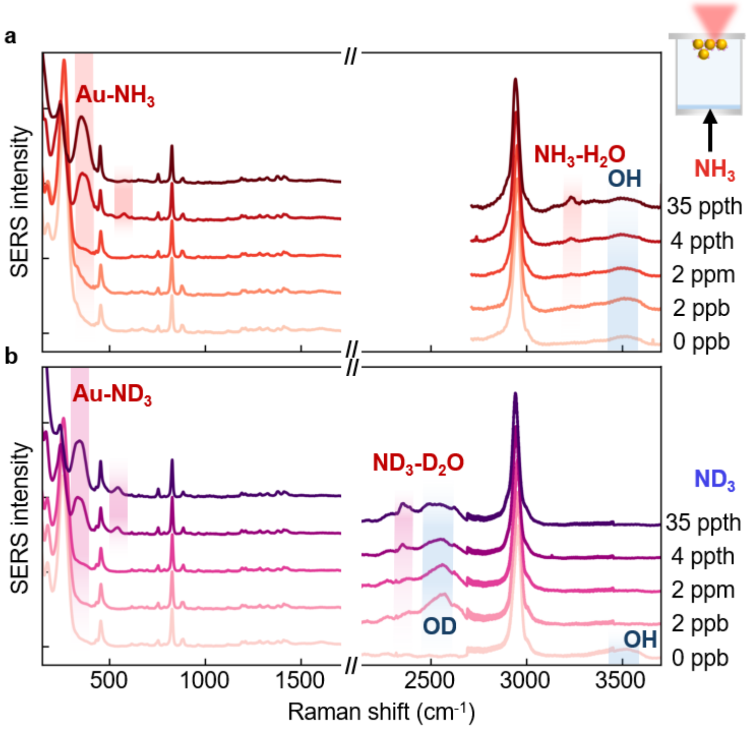

Figure S7. Comparison of NH_3 and ND_3 in headspace. SERS spectra for comparable headspace concentrations of **(a)** NH_3 and **(b)** ND_3, showing the peaks shifts of the Au-N and N-H peaks on deuteration.

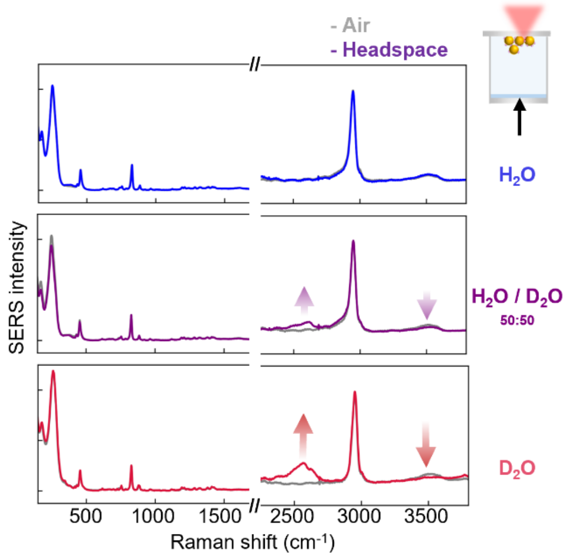

Figure S8. Exchange of surface water with D_2O in the headspace. No change in the MLagg SERS is seen for the headspace above H_2O (blue) compared to in air (grey). When D_2O is introduced to the headspace, the surface water on the MLagg is exchanged partially for H_2O-D_2O mixture (purple) and completely for D_2O (red).

`

To further understand the peaks, <1 M solutions of ammonia in D_2O are prepared from the 28% NH_3 stock solution (to have a majority D atoms) and left several weeks for H-D exchange to occur. Comparison of headspace sensing with these deuterated solutions shows a redshift in the Au-N peak by ~20 cm^{-1}, as expected for the heavier D atoms of the ND_3 (Fig. 2F, S7) which confirms this peak is due to the ammonia molecules. In contrast, there is an 880 cm^{-1} redshift for the ND_3-D_2O peaks, as the D atoms are directly involved in the bonds giving rise to the SERS peaks. Au-N and N-D peaks appear at similar headspace concentrations. When moving from air to the deuterated headspace, exchange of surface water for D_2O also occurs. This is observed in the headspace of pure D_2O and H_2O-D_2O mixtures and so is not driven by the presence of ammonia (Fig. S8). Due to their different partial pressures, the headspace concentration of water vapour is 31 ppth for pure H_2O and 27 ppth for pure D_2O, which is sufficient to drive the H_2O-D_2O exchange on the nanoparticle surface. This suggests that control of the solvent present in the headspace can be used to mediate MLagg-analyte interactions.

Similar effects are seen for SERS measurements of MLaggs in 1 M NH_3 solutions in H_2O and D_2O (immersed for 30 minutes) at low wavenumbers (Au-N peaks at 380 and 356 cm^{-1} respectively, but with key differences in other regions of the spectrum (Fig. S9A,B). No intermediate Au-OH is formed, all N-H peaks are shifted to higher wavenumbers, and the sharp N-H line corresponding to NH_3-NH_3 does not appear, even at high concentrations. This implies that when present in solution, interactions of the NH_3 molecules with the higher concentration of H_2O or D_2O molecules disrupt H-bonding between NH_3 molecules, as bulk water bridges the nanoparticle gaps rather than existing as a monolayer (Fig. S9C). Peak positions and ratios in these spectra agree closely with peak positions of previously reported SERS and Raman spectra of NH_3 solutions (unlike the headspace spectra) (*24*, *25*). This supports assignment of the 3293 cm^{-1} peak to the N-H stretch from NH_3-NH_3 interactions, present in headspace but not solution conditions.

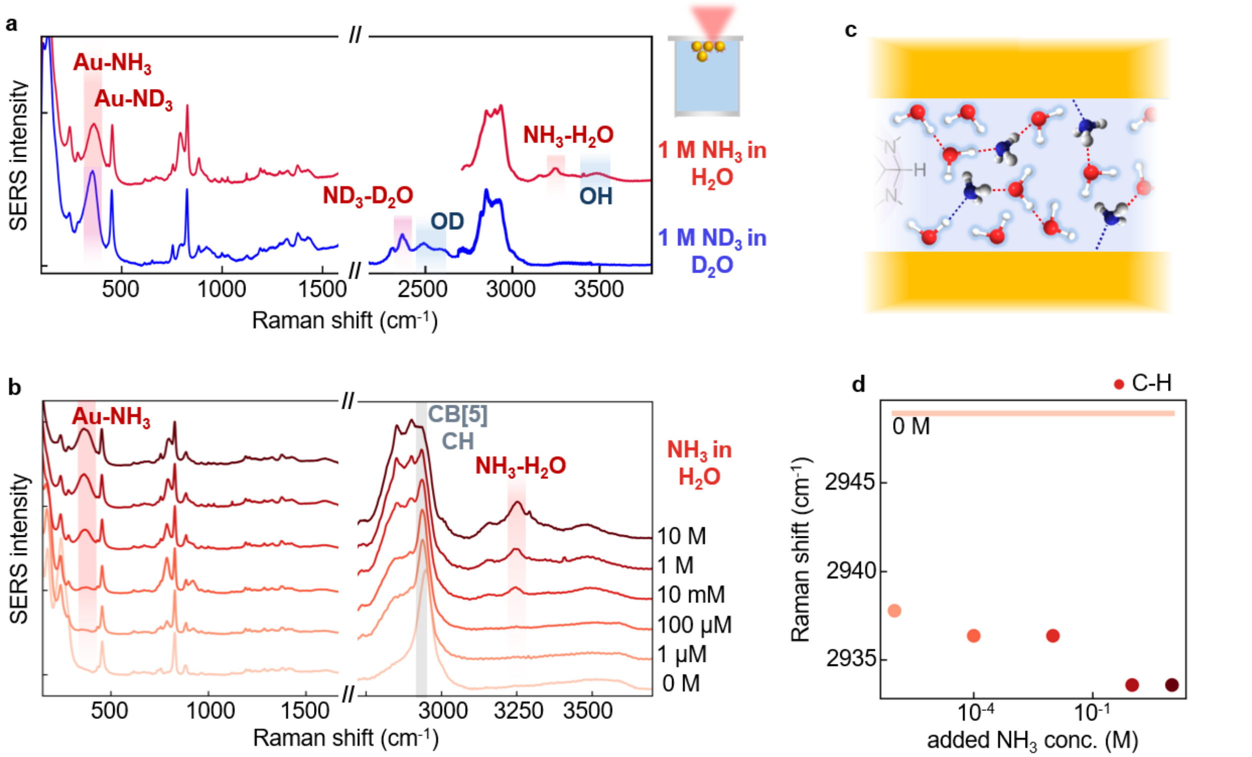

Figure S9. SERS of NH_3 solutions. (a) SERS of 1 M NH_3 solution in H_2O (red) and D_2O (blue). Peaks at high wavenumber are slightly shifted from their positions with headspace sensing due to different solvation of the ammonia molecules. **(b)** Similar peak evolution is seen over a concentration range of NH_3 in solution as in headspace, but with minimal appearance of the N-H line from NH_3-NH_3, even at high concentration (10 M). **(c)** Sketch of different ammonia-MLagg interactions when in solution. **(d)** Extracted peak position of C-H CB[5] peak, showing shifted already at 1μM added NH_3 solution.

Direct comparisons can be made between vapour and liquid sensing, using the SERS spectra of ammonia measured in 100 μM solution, where clear N-H and Au-N peaks are apparent, to the same solution in the headspace. From the headspace, the observed Au-N peak is 5 times larger (normalised to the 826 cm^{-1} CB[5] peak) compared to in solution. The height of the N-H peak from NH_3-H_2O is comparable. This highlights that water in solution-based sensing competes for MLagg nanogap binding sites and reduces Au-N interactions (by ~80%). We note the water monolayer is dynamic (as demonstrated by the water-D_2O exchange) which may play a crucial role in the binding dynamics of VOCs in these nanogaps. This also highlights that humidity control is vital for control of MLagg-VOC interactions and thus reliable SERS sensing of VOCs.

`

Section S6: Alternative surface sensitisations for ammonia sensing

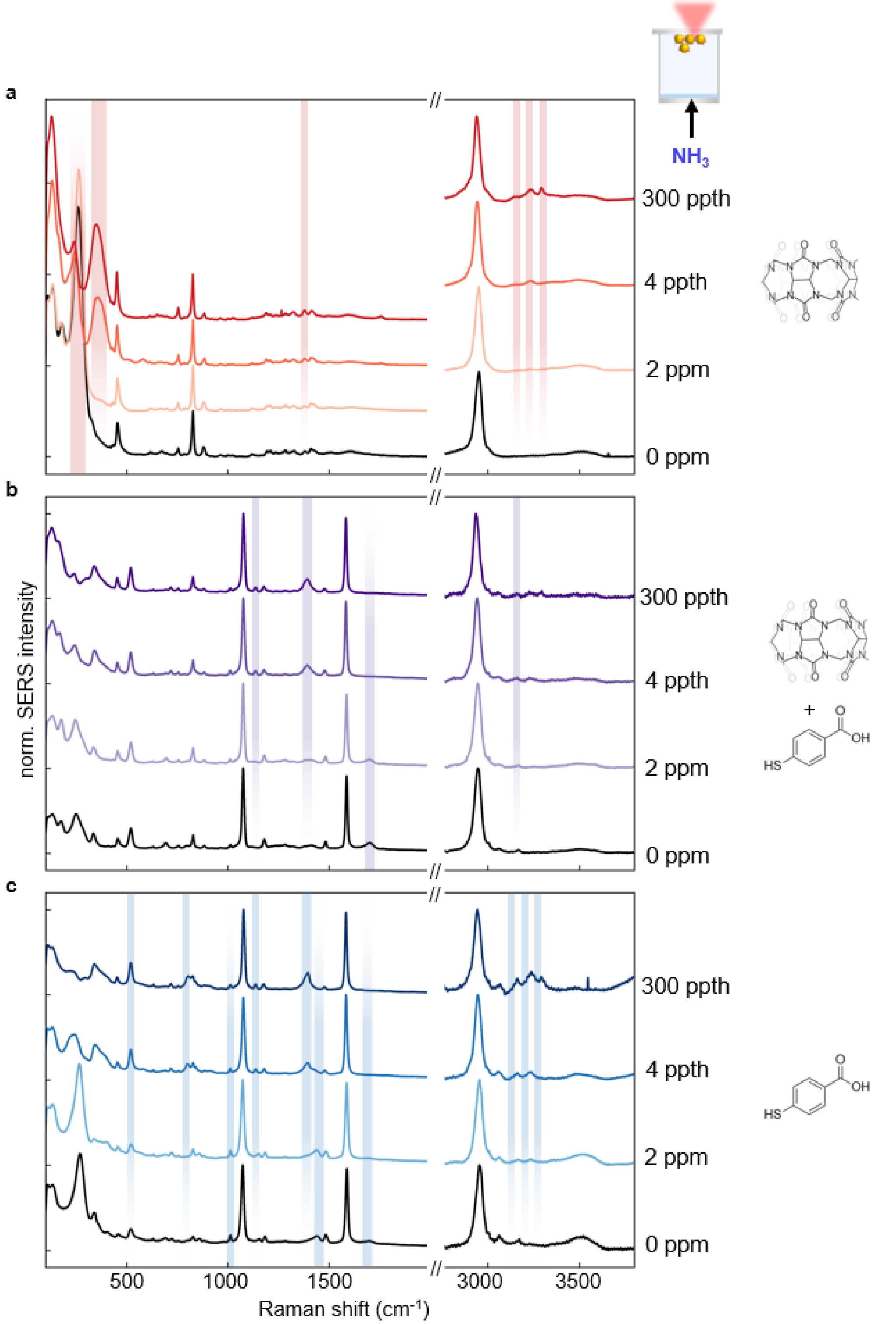

Figure S10. NH_3 concentration series comparison for CB[5] and MBA MLaggs. Headspace SERS for MLaggs scaffolded with **(a)** CB[5], **(b)** CB[5] and MBA and **(c)** MBA. Peak changes related to deprotonation are seen in the MBA MLaggs with increasing NH_3 concentration.

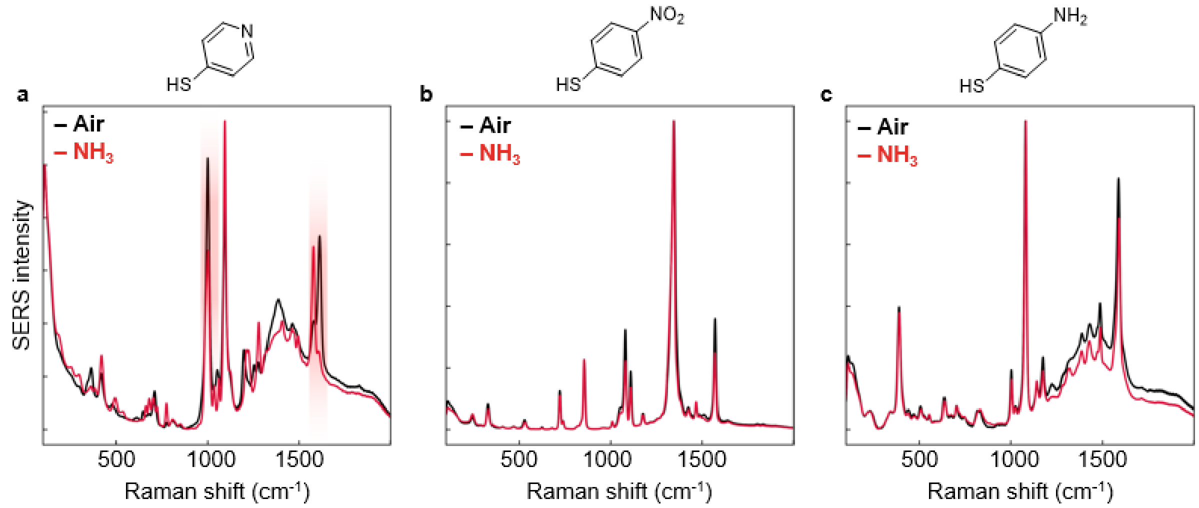

Figure S11. NH_3 response of different thiol scaffolds. (a) Peaks of the MPY MLagg change related to deprotonation and hydrogen bonding effects in 300 ppth NH_3. MLaggs scaffolded with **(b)** NTP and **(c)** ATP show weak peak shifts and intensity changes when in NH_3.

Through the plasma cleaning and re-scaffolding step, there is the opportunity to tune the nanogap chemistry of these MLaggs through choice of re-scaffolding ligand. Thiols are a particularly effective choice, as the thiol group binds strongly to Au and removes the gold oxide after plasma cleaning, without HCl (*38*). This is demonstrated here using 4-mercaptobenzoic acid (MBA), which is commonly used as a SERS pH indicator due to changes in its SERS spectrum as the carboxylic acid group is protonated and deprotonated (*39*). Comparing three MLagg samples (CB[5] scaffolded, CB[5] scaffolded then immersed in MBA and dried, and MBA scaffolded), changes in the SERS spectra of the MLaggs on exposure to NH_3 can be compared (Figure 3a). For CB[5]-MBA (purple) and MBA (blue) MLaggs the direct binding of NH_3 to the Au surface is inhibited due to the dense packing of thiol molecules.

Besides their reduced Au-N signals, with MBA a decrease in the 1700 cm^{-1} peak (COOH stretch) coupled with an increase in the 1400 cm^{-1} peak (COO^- stretch) is observed with NH_3, due to deprotonation of the MBA carboxylic acid functional group. In the high wavenumber region, changes are also seen in the MBA ~3050 and 3150 cm^{-1} peaks (O-H and C-H stretches) (*40*) and also shifts in the C-H peaks of MBA and CB[5]. The water O-H stretch is stronger in the pure MBA MLagg, but only due to normalisation by the C-H line which is less intense for MBA (C-H line has 4x fewer counts than for CB[5]). With MBA, the effects of ammonia can be observed in SERS down to ~2 ppm (Fig. S10), but likely this is purely due to non-specific (de)protonation and thus other analytes which change MBA protonation cannot be clearly distinguished.

The sensitization of the MLagg surface thus depends on the ability of an analyte (e.g. NH_3) to interact either with the scaffolding molecule, the AuNP surface, or the water monolayer. The choice of both the solvent in the gaps, and the scaffold molecule, impact the detection of NH_3 vapour. We demonstrate this by scaffolding an MLagg with the hydrophobic molecule biphenylthiol (BPT), which eliminates water from the nanogaps (seen by the absence of O-H lines ~3500 cm^{-1}, black line Fig.3b). This gives only minimal shifts in the BPT peaks (<2 cm^{-1}) and intensity ratio changes in the presence of NH_3 vapour (black to green, Fig.3b). The BPT molecules also prevent NH_3 molecules from reaching the nanoparticle surface, as no Au-N peak appears (due to tighter packing of the BPT scaffold molecules). Similarly, at high wavenumbers, minimal changes in BPT peaks are seen, and no N-H peaks appear. A number of other thiol scaffolds were also tested for ammonia sensing (Fig. S11), which either show large changes in the SERS spectrum due to protonation and hydrogen bonding as for MBA (eg. mercaptopyridine MPY with C=C peak shifts from 1610 cm^{-1} to 1580 cm^{-1}, changes in intensity ratio of ring modes at 1000 cm^{-1} and 1100 cm^{-1}) (*51*), or only slight peak intensity changes and peak shifts similar to BPT (aminothiophenol ATP, and nitrothiophenol NTP).

The nanogap solvent environment can also be altered by soaking CB[5]-scaffolded MLaggs in DMSO for 30 minutes. The DMSO interacts strongly with the AuNP surfaces and replaces most of the surface water even when the MLagg is removed from DMSO and dried (black Fig.3c) (*30*, *41*). On subsequent exposure to NH_3, the lack of water monolayer and blocking of the AuNP surfaces by DMSO changes all surface interactions (green Fig.3c). NH_3 displaces some DMSO (70% reduction in 670 and 1020 cm^{-1} DMSO peaks) while the Au-N peak is much (ten-fold) weaker than without DMSO. The high wavenumber ammonia spectrum is also changed, with a weaker NH_3-DMSO peak. Since DMSO is aprotic, NH_3 no longer ionises and no OH^- peaks are seen.

`

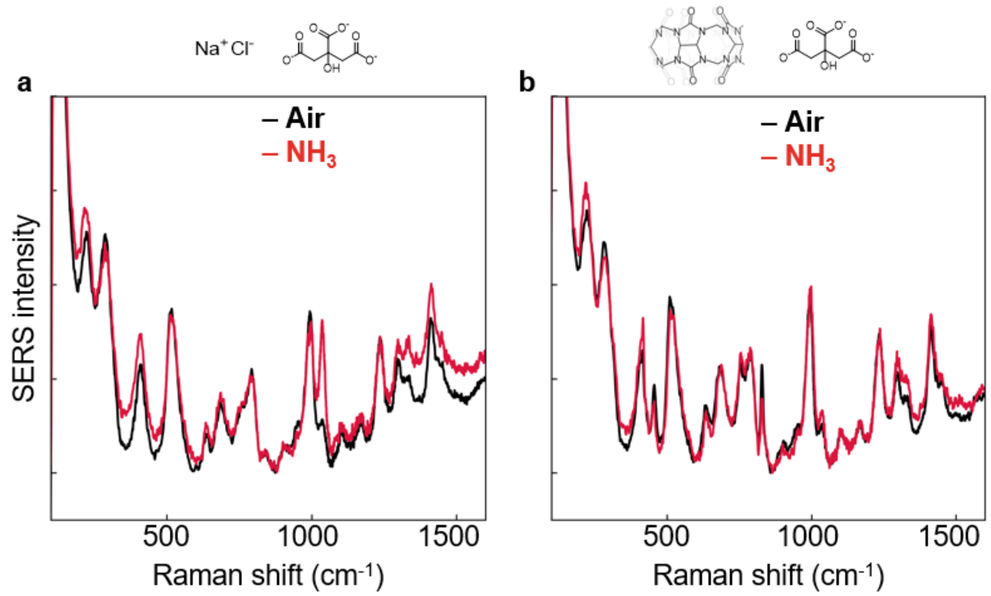

Figure S12. NH_3 response for MLaggs without cleaning and rescaffolding. MLaggs aggregated with **(a)** NaCl and **(b)** CB[5], and then used for 300 ppth NH_3 sensing (without plasma cleaning and re-scaffolding) have citrate and surfactants on the AuNP surfaces which blocks Au-N interactions.

Table S2. VOC headspace concentration calculations.

Volatile compound	P at ~20°C (Pa)	x	P (ppm)
Acetone	25544 (*52*)	1	252000
Isopropanol	4444 (*53*)	1	43900
Ethanol	8200 (*54*)	1	80900
Methanol	16850 (*54*)	1	1660000
Cyclopentanone	1560 (*55*)	1	15400

`

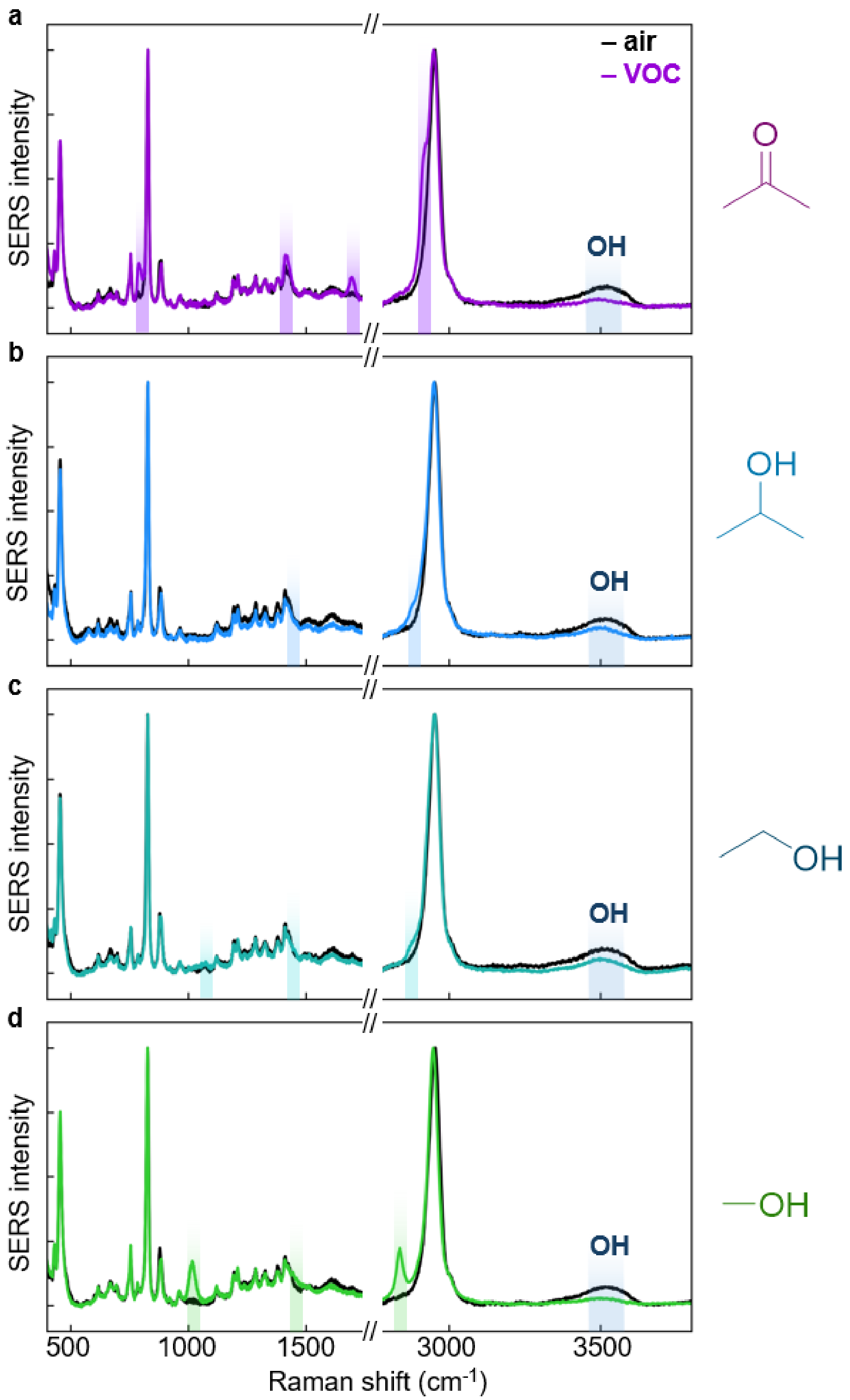

Figure S13. SERS sensing of VOCs using CB[5] scaffolded MLaggs. Low and high wavenumber SERS spectra for MLaggs in air (black lines) and in the headspace of four different VOCS at undiluted saturation concentration (part per thousand = ppth): (**a**) acetone (252 ppth), (**b**) IPA (44 ppth), (**c**) ethanol (81 ppth) and (**d**) methanol (1660 ppth), showing the appearance of peaks characteristic to each molecule (highlighted), and a decrease in the O-H line.

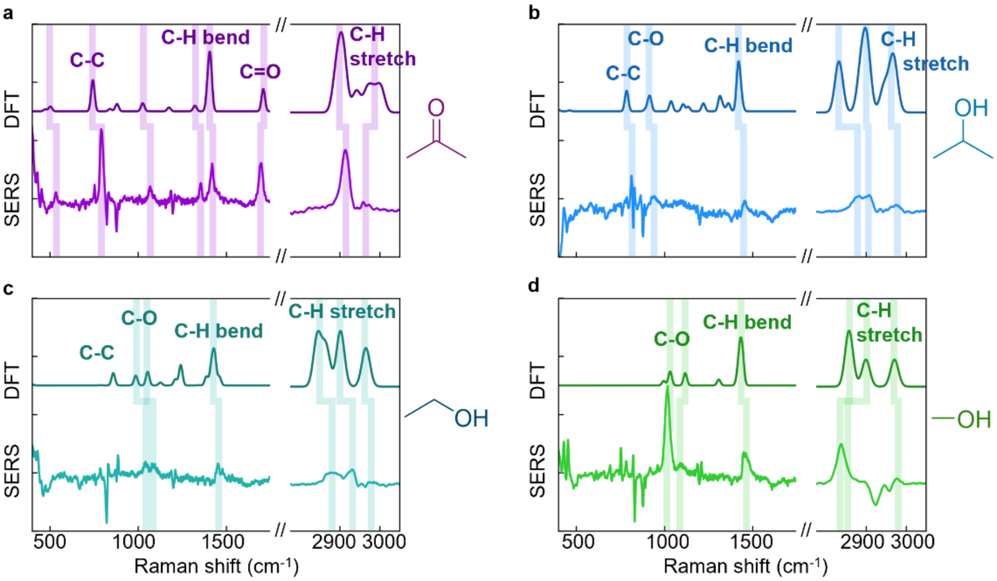

Figure S14. DFT calculations for VOCs. Comparison of DFT calculations of Raman activities for **(a)** acetone, **(b)** IPA, **(c)** ethanol and **(d)** methanol to the SERS spectrum, after reference in air subtracted. Shaded lines indicate likely peak assignments.

Section S7: DFT calculations

Density functional theory (DFT) calculations with the B3LYP and def2tzvp basis set were used to calculate Raman spectra for each VOC molecule. Molecule structures were initially optimized using Chem3D (Revvity Signals Software, Inc.), and then Gaussian 09 used for further optimization and to calculate the frequency and intensity of the Raman modes.

Spectra are plotted using Gaussian lineshapes with a width of 4 cm^{-1} and the Raman shifts were scaled by 0.975 (as conventional) to provide a better match to experimental SERS spectra.

For comparison to experimental data, the SERS spectrum in air was subtracted from the VOC SERS spectra. For the high wavenumber region, the CB[5] C-H peak shift was first applied to the SERS spectrum in air prior to subtraction, in order to minimise effects from CB[5] C-H peak shifts.

Section S8: Re-scaffolding sensitization for other VOCs

Thiolated scaffolds similarly modify MLagg interactions with these VOCs. For BPT-scaffolded MLaggs, sensing of acetone and methanol results in small but repeatable shifts of the major BPT lines (Fig. S15). A consistent redshift of the 1280 cm^{-1} peak (C-C) is found for both VOCs, but the effect on the 1080 cm^{-1} peak (ring breathing) is analyte dependent, blueshifting for methanol but slightly narrowing for acetone. Slightly larger effects are seen for methanol in a triphenylthiol (TPT) scaffolded MLagg compared to BPT (Fig.S14). This implies that such VOC molecules diffuse into densely-packed molecular layers in the nanogaps and perturb their vibrations. Previously, shifts have been related to BPT conformations and ring twists (*56*), which can be modified in the presence of VOC molecules.

Cysteamine scaffolded MLaggs have a specific response to ketones over alcohols. Bridging S-S interactions are formed between neighbouring cysteamine molecules in the nanogaps, as seen in SERS spectra (*42*). On exposure to VOCs, a decrease in S-S peak is observed here for ketones (acetone and cyclopentanone), but not for alcohols (ethanol and methanol) (Fig. S16a). This is due to hydrogen bonding interactions between the cysteamine NH_2 and the ketone C=O (*43*), which forces apart and weakens the S-S links (Fig. S16b). The reduction in S-S intensity is related to the number of ketone molecules (30% reduction for 15 ppth cyclopentanone and 65% reduction for 252 ppth acetone). This demonstrates that through the choice of nanogap scaffold, selectivity for VOCs with particular functional groups can be achieved.

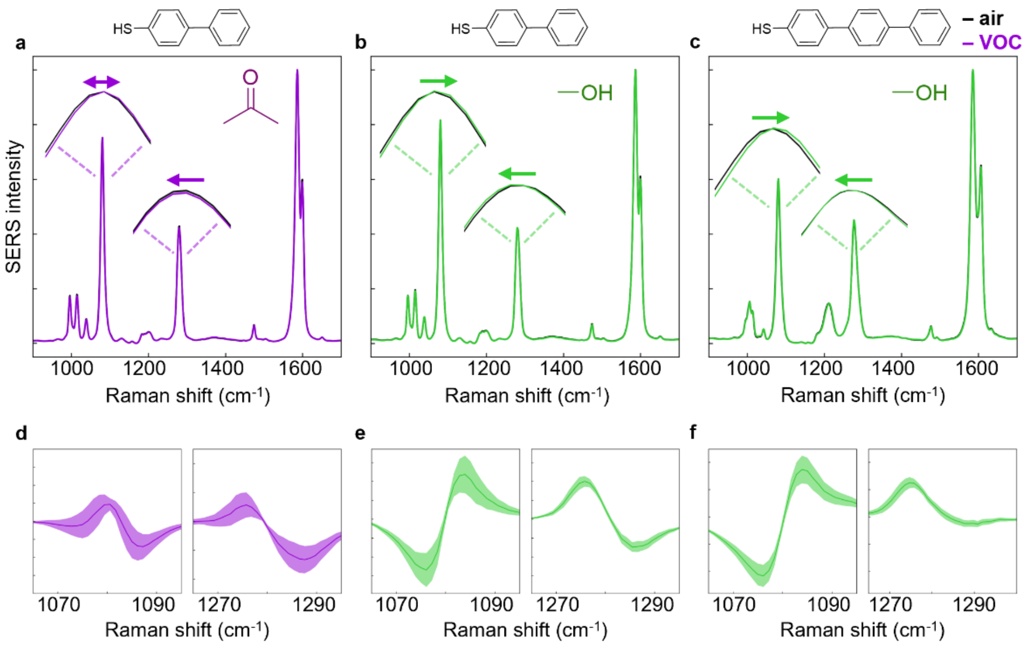

Figure S15. VOC sensing using BPT and TPT scaffolded MLaggs. (a-c) Subtle peak shifts are seen in the peaks of the BPT and TPT spectra when in pure acetone or methanol headspace compared to in air. **(d-f)** Subtraction of the headspace and air spectra reveal a consistent redshift of the 1280 cm^{-1} peak for all samples, but the 1080 cm^{-1} peak clearly blueshifts for methanol in both BPT and TPT scaffolded MLaggs and slightly narrows for acetone in BPT MLaggs. Shading represents the standard deviation of 5 repeats.

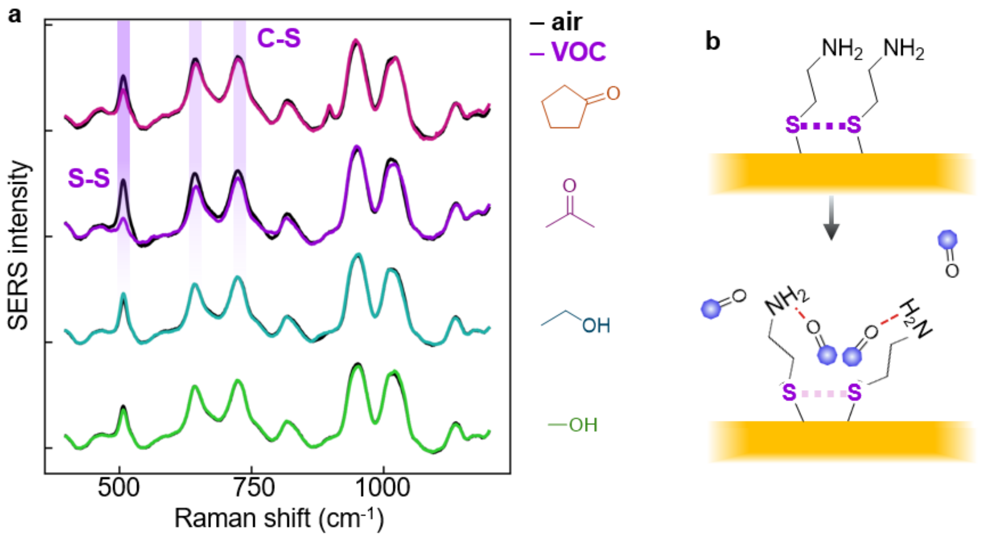

Figure S16. Selective VOC interactions with cysteamine scaffold. (a) SERS spectra for headspace measurements of ketones and alcohols, showing a decrease in the S-S peak only when in a ketone headspace (coloured line) compared to in air (black line). Saturated headspace concentrations above pure solutions are cyclopentanone (15 ppth), acetone (252 ppth), ethanol (81 ppth) and methanol (1660 ppth). **(b)** Sketch of possible interactions, as presence of VOCs with a carboxyl functional group (blue symbol) intercalates into cysteamine layer, forcing apart molecules and thus breaking S-S bonds between cysteamine molecules.

`

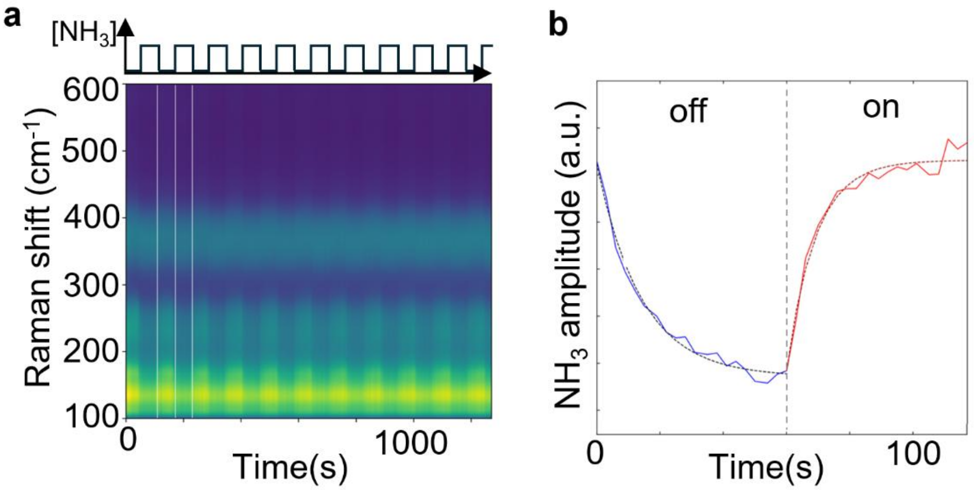

Figure S17. Dynamics of NH_3 binding. (a) SERS spectra of MLagg-CB[5] in gas flow of either N_2 alone, or N_2 bubbled through NH_3 solution, showing repeatable signal observed. **(b)** Average dynamics of NH_3 binding and unbinding observed in flow cell, giving decay time of 14.1s and rise time of 9.1s.

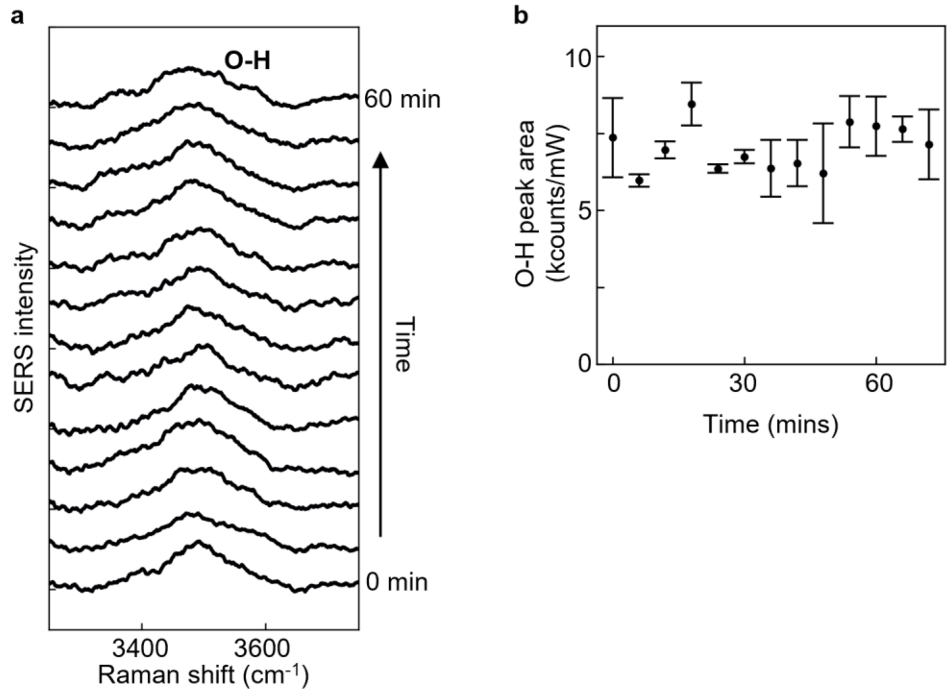

Figure S18. O-H peak measured at the same MLagg position over time. (a) Even after repeated laser exposure, the O-H peak intensity remains constant over 60 mins, plotted in **(b)**. Measurements made with 10% laser power and 10 s integration time, with the MLagg in 50 sccm N_2 flow.

REFERENCES

1. L. D. Lehman-McKeeman, "Absorption, distribution, and excretion of toxicants," in *Casarett & Doull's Essentials of Toxicology, Third Edition* (McGraw-Hill Education, 2015).

2. S. Li, A. Simonian, B. A. Chin, Sensors for agriculture and the food industry. *Electrochem. Soc. Interface* **19**, 41–46 (2010).

3. X. Zhou, Z. Xue, X. Chen, C. Huang, W. Bai, Z. Lu, T. Wang, Nanomaterial-based gas sensors used for breath diagnosis. *J. Mater. Chem. B* **8**, 3231–3248 (2020).

4. A. Sharma, R. Kumar, P. Varadwaj, Smelling the disease: Diagnostic potential of breath analysis. *Mol. Diagn. Ther.* **27**, 321–347 (2023).

5. F. Di Francesco, R. Fuoco, M. G. Trivella, A. Ceccarini, Breath analysis: Trends in techniques and clinical applications. *Microchem. J.* **79**, 405–410 (2005).

6. J. Dummer, M. Storer, M. Swanney, M. McEwan, A. Scott-Thomas, S. Bhandari, S. Chambers, R. Dweik, M. Epton, Analysis of biogenic volatile organic compounds in human health and disease. *Trends Analyt. Chem.* **30**, 960–967 (2011).

7. J. Obermeier, P. Trefz, J. Happ, J. K. Schubert, H. Staude, D.-C. Fischer, W. Miekisch, Exhaled volatile substances mirror clinical conditions in pediatric chronic kidney disease. *PLOS ONE* **12**, e0178745 (2017).

8. C. Turner, P. Spanel, D. Smith, A longitudinal study of ammonia, acetone and propanol in the exhaled breath of 30 subjects using selected ion flow tube mass spectrometry, SIFT-MS. *Physiol. Meas.* **27**, 321–337 (2006).

9. S. Davies, P. Spanel, D. Smith, Quantitative analysis of ammonia on the breath of patients in end-stage renal failure. *Kidney Int.* **52**, 223–228 (1997).

10. P. C. Moura, M. Raposo, V. Vassilenko, Breath volatile organic compounds (VOCs) as biomarkers for the diagnosis of pathological conditions: A review. *Biomed. J.* **46**, 100623 (2023).

11. C. Pinheiro, C. M. Rodrigues, T. Schäfer, J. G. Crespo, Monitoring the aroma production during wine–must fermentation with an electronic nose. *Biotechnol. Bioeng.* **77**, 632–640 (2002).

12. P. Ivanov, E. Llobet, A. Vergara, M. Stankova, X. Vilanova, J. Hubalek, I. Gracia, C. Cané, X. Correig, Towards a micro-system for monitoring ethylene in warehouses. *Sens. Actuators B Chem.* **111-112**, 63–70 (2005).

13. C. Baratto, G. Faglia, M. Pardo, M. Vezzoli, L. Boarino, M. Maffei, S. Bossi, G. Sberveglieri, Monitoring plants health in greenhouse for space missions. *Sens. Actuators B Chem.* **108**, 278–284 (2005).

14. K. E. Wyer, D. B. Kelleghan, V. Blanes-Vidal, G. Schauberger, T. P. Curran, Ammonia emissions from agriculture and their contribution to fine particulate matter: A review of implications for human health. *J. Environ. Manage.* **323**, 116285 (2022).

15. R. Zaffaroni, D. Ripepi, J. Middelkoop, F. M. Mulder, Gas chromatographic method for in situ ammonia quantification at parts per billion levels. *ACS Energy Lett.* **5**, 3773–3777 (2020).

16. N. H. Snow, Flying high with sensitivity and selectivity: GC–MS to GC–MS/MS. *LCGC N. Am.* **39**, 61–67 (2021).

17. M. Libardoni, P. T. Stevens, J. H. Waite, R. Sacks, Analysis of human breath samples with a multi-bed sorption trap and comprehensive two-dimensional gas chromatography (GC×GC). *J. Chromatogr. B Analyt. Technol. Biomed. Life Sci.* **842**, 13–21 (2006).

18. W. Miekisch, J. K. Schubert, From highly sophisticated analytical techniques to life-saving diagnostics: Technical developments in breath analysis. *Trends Analyt. Chem.* **25**, 665–673 (2006).

19. D. Kwak, Y. Lei, R. Maric, Ammonia gas sensors: A comprehensive review. *Talanta* **204**, 713–730 (2019).

20. M. J. Lefferts, M. R. Castell, Ammonia breath analysis. *Sens. Diagn.* **1**, 955–967 (2022).

21. N. Dennler, D. Drix, T. P. A. Warner, S. Rastogi, C. D. Casa, T. Ackels, A. T. Schaefer, A. Van Schaik, M. Schmuker, High-speed odor sensing using miniaturized electronic nose. *Sci. Adv.* **10**, eadp1764 (2024).

22. C. L. Haynes, A. D. McFarland, R. P. Van Duyne, Surface-enhanced Raman spectroscopy. *Anal. Chem.* **77**, 338A–346A (2005).

23. J. Langer, D. J. de Aberasturi, J. Aizpurua, R. A. Alvarez-Puebla, B. Auguié, J. J. Baumberg, G. C. Bazan, S. E. J. Bell, A. Boisen, A. G. Brolo, J. Choo, D. Cialla-May, V. Deckert, L. Fabris, K. Faulds, F. J. G. de Abajo, R. Goodacre, D. Graham, A. J. Haes, C. L. Haynes, C. Huck, T. Itoh, M. Käll, J. Kneipp, N. A. Kotov, H. Kuang, E. C. L. Ru, H. K. Lee, J. F. Li, X. Y. Ling, S. A. Maier, T. Mayerhöfer, M. Moskovits, K. Murakoshi, J. M. Nam, S. Nie, Y. Ozaki, I. Pastoriza-Santos, J. Perez-Juste, J. Popp, A. Pucci, S. Reich, B. Ren, G. C. Schatz, T. Shegai, S. Schlücker, L. L. Tay, K. G. Thomas, Z. Q. Tian, R. P. van Duyne, T. Vo-Dinh, Y. Wang, K. A. Willets, C. Xu, H. Xu, Y. Xu, Y. S. Yamamoto, B. Zhao, L. M. Liz-Marzán, Present and future of surface-enhanced Raman scattering. *ACS Nano* **14**, 28–117 (2020).

24. Y. Liu, T. Asset, Y. Chen, E. Murphy, E. O. Potma, I. Matanovic, D. A. Fishman, P. Atanassov, Facile all-optical method for in situ detection of low amounts of ammonia. *iScience* **23**, 101757 (2020).

25. T. Ujike, Y. Tominaga, Raman spectral analysis of liquid ammonia and aqueous solution of ammonia. *J. Raman Spectrosc.* **33**, 485–493 (2002).

26. C. Yang, D. Ezendeeva, T. Yu, G. Magnotti, Temperature dependent Raman spectra of ammonia ranging from 3150 cm^{-1} to 3810 cm^{-1} for combustion applications. *Opt. Express* **29**, 33234–33244 (2021).

27. D. B. Grys, M. Niihori, R. Arul, S. M. Sibug-Torres, E. W. Wyatt, B. de Nijs, J. J. Baumberg, Controlling atomic-scale restructuring and cleaning of gold nanogap multilayers for surface-enhanced raman scattering sensing. *ACS Sens.* **8**, 2879–2888 (2023).

28. S. M. Sibug-Torres, D.-B. Grys, G. Kang, M. Niihori, E. Wyatt, N. Spiesshofer, A. Ruane, B. De Nijs, J. J. Baumberg, In situ electrochemical regeneration of nanogap hotspots for continuously reusable ultrathin SERS sensors. *Nat. Commun.* **15**, 2022 (2024).

29. M. Niihori, T. Földes, C. A. Readman, R. Arul, D.-B. Grys, B. de Nijs, E. Rosta, J. J. Baumberg, SERS sensing of dopamine with Fe(III)-sensitized nanogaps in recleanable AuNP monolayer films. *Small* **19**, e2302531 (2023).

30. E. W. Wyatt, S. M. Sibug-Torres, R. Arul, M. Niihori, T. Jones, J. W. Beattie, B. de Nijs, J. J. Baumberg, Tracking and controlling monolayer water in gold nanogaps using extreme plasmonic spectroscopy. *Small* **21**, e07013 (2025).

31. C. S. L. Koh, H. K. Lee, X. Han, H. Y. F. Sim, X. Y. Ling, Plasmonic nose: integrating the MOF-enabled molecular preconcentration effect with a plasmonic array for recognition of molecular-level volatile organic compounds. *Chem. Commun.* **54**, 2546–2549 (2018).

32. Y. Obeidat, A. M. Rawashdeh, A. Hammoudeh, R. Al-Assi, A. Dagamseh, Q. Qananwah, Acetone sensing in liquid and gas phases using cyclic voltammetry. *Sci. Rep.* **12**, 11010 (2022).

33. A. C. A. de Vooys, M. F. Mrozek, M. T. M. Koper, R. A. van Santen, J. A. R. van Veen, M. J. Weaver, The nature of chemisorbates formed from ammonia on gold and palladium electrodes as discerned from surface-enhanced Raman spectroscopy. *Electrochem. Commun.* **3**, 293–298 (2001).

34. Y. Liu, E. Murphy, E. O. Potma, I. Matanovic, D. A. Fishman, P. Atanassov, Protocol for rapid ammonia detection via surface-enhanced Raman spectroscopy. *STAR Protoc.* **2**, 100599 (2021).

35. N. A. Rogozhnikov, A study of ammonia adsorption on gold face (111). *Mater. Today Proc.* **31**, 473–475 (2020).

36. J. H. K. Pfisterer, F. Nattino, U. E. Zhumaev, M. Breiner, J. M. Feliu, N. Marzari, K. F. Domke, Role of OH intermediates during the Au oxide electro-reduction at low pH

elucidated by electrochemical surface-enhanced Raman spectroscopy and implicit solvent density functional theory. *ACS Catal.* **10**, 12716–12726 (2020).

37. D. Grys, R. Chikkaraddy, M. Kamp, O. A. Scherman, J. J. Baumberg, B. De Nijs, Eliminating irreproducibility in SERS substrates. *J. Raman Spectrosc.* **52**, 412–419 (2021).

38. Y. Xue, X. Li, H. Li, W. Zhang, Quantifying thiol–gold interactions towards the efficient strength control. *Nat. Commun.* **5**, 4348 (2014).

39. F. C. Marques, R. S. Alves, D. P. Dos Santos, G. F. S. Andrade, Surface-enhanced Raman spectroscopy of one and a few molecules of acid 4-mercaptobenzoic in AgNP enabled by hot spots generated by hydrogen bonding. *Phys. Chem. Chem. Phys.* **24**, 27449–27458 (2022).

40. K. S. Bai, Raman spectra of some carboxylic acids. *Proc. Indian Acad. Sci. Sect. A* **11**, 212–228 (1940).

41. K. I. Oh, K. Rajesh, J. F. Stanton, C. R. Baiz, Quantifying hydrogen-bond populations in dimethyl sulfoxide/water mixtures. *Angew. Chem. Int. Ed. Engl.* **56**, 11375–11379 (2017).

42. H. Häkkinen, The gold–sulfur interface at the nanoscale. *Nat. Chem.* **4**, 443–455 (2012).

43. Z. Xie, M. V. Ramakrishnam Raju, P. K. Adhihetty, X.-A. Fu, M. H. Nantz, Effect of thiol molecular structure on the sensitivity of gold nanoparticle-based chemiresistors toward carbonyl compounds. *Sensors* **20**, 7024 (2020).

44. N. Kim, M. R. Thomas, M. S. Bergholt, I. J. Pence, H. Seong, P. Charchar, N. Todorova, A. Nagelkerke, A. Belessiotis-Richards, D. J. Payne, A. Gelmi, I. Yarovsky, M. M. Stevens, Surface enhanced Raman scattering artificial nose for high dimensionality fingerprinting. *Nat. Commun.* **11**, 207 (2020).

45. K. N. Han, M. C. Fuerstenau, Factors influencing the rate of dissolution of gold in ammoniacal solutions. *Int. J. Miner. Process.* **58**, 369–381 (2000).

46. J. Huang, B. de Nijs, S. Cormier, K. Sokolowski, D.-B. Grys, C. A. Readman, S. J. Barrow, O. A. Scherman, J. J. Baumberg, Plasmon-induced optical control over dithionite-mediated chemical redox reactions. *Faraday Discuss.* **214**, 455–463 (2019).

47. W. H. Skinner, R. L. Sala, K. Sokolowski, I. Blein-Dezayes, N. S. Potter, S. Mosca, B. Gardner, J. J. Baumberg, P. Matousek, O. A. Scherman, N. Stone, An all-in-one nanoheater and optical thermometer fabricated from fractal nanoparticle assemblies. *ACS Nano* **19**, 13779–13789 (2025).

48. C. S. Cragoe, C. H. Meyers, C. S. Taylor, The vapour pressure of ammonia. *J. Am. Chem. Soc.* **42**, 206–229 (1920).

49. Q. Shi, P. Davidovits, J. T. Jayne, D. R. Worsnop, C. E. Kolb, Uptake of gas-phase ammonia. 1. Uptake by aqueous surfaces as a function of pH. *J. Phys. Chem. A* **103**, 8812–8823 (1999).

50. S. L. Clegg, P. Brimblecombe, Solubility of ammonia in pure aqueous and multicomponent solutions. *J. Phys. Chem.* **93**, 7237–7248 (1989).

51. N. Wattanavichean, E. Casey, R. J. Nichols, H. Arnolds, Discrimination between hydrogen bonding and protonation in the spectra of a surface-enhanced Raman sensor. *Phys. Chem. Chem. Phys.* **20**, 866–871 (2018).

52. W. A. Feslings, A. Durban, The vapor pressures, densities, and some derived quantities for acetone. *J. Am. Chem. Soc.* **48**, 2885–2893 (1926).

53. G. S. Parks, B. Barton, Vapor pressure data for isopropyl alcohol and tertiary butyl alcohol. *J. Am. Chem. Soc.* **50**, 24–26 (1928).

54. D. Ambrose, C. H. S. Sprake, Thermodynamic properties of organic oxygen compounds XXV. Vapour pressures and normal boiling temperatures of aliphatic alcohols. *J. Chem. Thermodyn.* **2**, 631–645 (1970).

55. S. W. Benson, G. B. Kistiakowsky, The photochemical decomposition of cyclic ketones. *J. Am. Chem. Soc.* **64**, 80–86 (1942).

56. D. Kos, G. Di Martino, A. Boehmke, B. de Nijs, D. Berta, T. Földes, S. Sangtarash, E. Rosta, H. Sadeghi, J. J. Baumberg, Optical probes of molecules as nano-mechanical switches. *Nat. Commun.* **11**, 5905 (2020).
